# Effect of Nasal Continuous Positive Airway Pressure vs Heated Humidified High-Flow Nasal Cannula on Feeding Intolerance in Preterm Infants With Respiratory Distress Syndrome: The ENTARES Randomized Clinical Trial

**DOI:** 10.1001/jamanetworkopen.2023.23052

**Published:** 2023-07-12

**Authors:** Francesco Cresi, Elena Maggiora, Gianluca Lista, Carlo Dani, Silvia M. Borgione, Elena Spada, Mattia Ferroglio, Enrico Bertino, Alessandra Coscia

**Affiliations:** 1 Neonatology and Neonatal Intensive Care Unit, Department of Public Health and Pediatrics, University of Turin, Turin, Italy; 2 Neonatal Pathology and Neonatal Intensive Care Unit, Vittore-Buzzi Children’s Hospital, Milan, Italy; 3 Division of Neonatology, Careggi University Hospital of Florence, Florence, Italy; 4 Fondazione IRCCS (Istituto di Ricovero e Cura a Carattere Scientifico) Ca’ Granda Ospedale Maggiore Policlinico, Neonatology and Neonatal Intensive Care Unit, Milan, Italy

## Abstract

**Importance:**

Respiratory distress syndrome and feeding intolerance are common conditions that are often associated with preterm infants. Showing similar efficacy, nasal continuous positive airway pressure (NCPAP) and heated humidified high-flow nasal cannula (HHHFNC) are the most widespread noninvasive respiratory support (NRS) in neonatal intensive care units, but their effect on feeding intolerance is unknown.

**Objective:**

To evaluate the effect of NCPAP vs HHHFNC on high-risk preterm infants with respiratory distress syndrome.

**Design, Setting, and Participants:**

This multicenter randomized clinical trial involved infants who were born in 1 of 13 neonatal intensive care units in Italy between November 1, 2018, and June 30, 2021. Preterm infants with a gestational age of 25 to 29 weeks, who were suitable for enteral feeding and who proved to be medically stable on NRS for at least 48 hours were enrolled in the study within the first week of life and randomized to receive either NCPAP or HHHFNC. Statistical analysis was performed according to the intention-to-treat approach.

**Intervention:**

NCPAP or HHHFNC.

**Main Outcomes and Measures:**

The primary outcome was the time to full enteral feeding (FEF), defined as an enteral intake of 150 mL/kg per day. Secondary outcomes were the median daily increment of enteral feeding, signs of feeding intolerance, effectiveness of the assigned NRS, peripheral oxygen saturation (SpO_2_)–fraction of inspired oxygen (FIO_2_) ratio at changes of NRS, and growth.

**Results:**

Two-hundred forty-seven infants (median [IQR] gestational age, 28 [27-29] weeks; 130 girls [52.6%]) were randomized to the NCPAP group (n = 122) or the HHHFNC group (n = 125). There were no differences in the primary and secondary nutritional outcomes between the 2 groups. The median time to reach FEF was 14 days (95% CI, 11-15 days) in the NCPAP group and 14 days (95% CI, 12-18 days) in the HHHFNC group, and similar results were observed in the subgroup of infants with less than 28 weeks’ gestation. On the first NRS change, higher SpO_2_–FIO_2_ ratio (median [IQR], 4.6 [4.1-4.7] vs 3.7 [3.2-4.0]; *P* < .001) and lower rate of ineffectiveness (1 [4.8%] vs 17 [73.9%]; *P* < .001) were observed in the NCPAP vs HHHFNC group.

**Conclusions and relevance:**

This randomized clinical trial found that NCPAP and HHHFNC had similar effects on feeding intolerance, despite different working mechanisms. Clinicians may tailor respiratory care by selecting and switching between the 2 NRS techniques on the basis of respiratory effectiveness and patient compliance, without affecting feeding intolerance.

**Trial Registration:**

ClinicalTrials.gov Identifier: NCT03548324

## Key Points

**Question:**

What is the effect of nasal continuous positive airway pressure (NCPAP) vs heated humidified high-flow nasal cannula (HHHFNC) on feeding intolerance in preterm infants?

**Findings:**

In this randomized clinical trial involving 247 preterm infants with respiratory distress who were randomized to either the NCPAP or HHHFNC arm, no differences were observed in the median time to achieve full enteral feeding (14 days in both groups) or in the evaluation of the signs of feeding intolerance. Better respiratory outcome was observed in the NCPAP group.

**Meaning:**

Findings of this study suggest that the type of noninvasive respiratory support does not directly influence feeding tolerance in preterm infants.

## Teaser

This randomized clinical trial compares 2 types of noninvasive respiratory support on the basis of daily enteral feeding, signs of feeding intolerance, treatment effectiveness, peripheral oxygen saturation levels, fraction of inspired oxygen levels, and growth and respiratory outcomes.

## Introduction

Respiratory distress syndrome (RDS) is a common issue in preterm infants.^1^ The use of noninvasive respiratory support (NRS) reduces the need for mechanical ventilation, the risk of death, intraventricular hemorrhage, and bronchopulmonary dysplasia in these infants.^2^

Preterm infants benefit from early initiation of enteral feeding^3^ but often experience symptoms of feeding intolerance and increased risk of necrotizing enterocolitis (NEC). Feeding intolerance is generally associated with the need to withhold enteral feeding, which prolongs the duration of parenteral nutrition, thus increasing the risk of infections and lengthening hospital stay.^4^ Along with RDS, feeding intolerance represents a major problem in preterm infants, and the coexistence of RDS and feeding intolerance presents neonatologists with a challenge.^5^

Noninvasive respiratory support could affect feeding intolerance through several mechanisms. First, part of the pressurized gas flows intended for the respiratory tract is transmitted to the gastrointestinal tract, causing its gaseous distension.^6^ Second, the gaseous distension of the intestine could generate mucosal inflammation due to cytokine release, as observed during NEC in basic science experiments.^7^ Moreover, the oxygen-enriched air mixture can induce potentially dangerous imbalances in the intestinal microbial flora,^8^ and the pressure induced by NRS can affect gastric emptying, gastroesophageal reflux, and mesenteric flows.^9,10,11^

The most widely used NRS modalities are nasal continuous positive airway pressure (NCPAP) and heated humidified high-flow nasal cannula (HHHFNC).^12^ Nasal continuous positive airway pressure has been recommended as a first-line treatment for RDS,^13,14^ particularly in extremely preterm infants, whereas HHHFNC has been found to be an effective alternative as postextubation support to prevent the resumption of mechanical ventilation.^15^ Some studies comparing NCPAP and HHHFNC have reported data on their possible effects on enteral nutrition. Although these data were conflicting,^5,15^ a recent meta-analysis suggested the benefits of HHHFNC vs NCPAP for feeding tolerance.^16^ Despite the effects of different types of NRS on nutrition and growth of very preterm infants, no randomized clinical trial has been specifically conducted to assess such outcomes.

Based on previous considerations, we hypothesized that HHHFNC may affect feeding intolerance to a lesser extent than NCPAP. We carried out this trial to evaluate the effect of NCPAP vs HHHFNC on high-risk preterm infants (gestational age <30 weeks) with respiratory distress syndrome.

## Methods

### Study Design and Setting

This randomized clinical trial involved infants who were born between November 1, 2018, and June 30, 2021, at 1 of 13 participating neonatal intensive care units (NICUs) across Italy. The study protocol (Supplement 1) was reviewed and approved by the institutional review board and ethics committee of the Azienda Ospedaliero Universitaria Città della Salute e della Scienza di Torino and by the ethics committee of all of the participating NICUs. Informed written consent was obtained from the parents of all participants. We followed the Consolidated Standards of Reporting Trials (CONSORT) reporting guideline.^17^

All infants with gestational age between 25 and 29 weeks, 7 days of life or less, suitability for enteral feeding (<75 mL/kg/d if already started), and RDS were eligible for the study. Respiratory distress syndrome was defined as respiratory failure requiring respiratory support, and surfactant was administered according to the European Consensus Guidelines on the Management of RDS.^2^ Infants with neurological or surgical diseases, sepsis, chromosomal abnormalities, and major malformations were excluded.

The intervention was the randomization of infants to either the NCPAP or HHHFNC arm. Since no conclusive data were available on the efficacy and safety of HHHFNC in extremely preterm infants as a first-intention approach at birth or after extubation, we decided to randomize only infants who proved to be medically stable for at least 48 hours on NRS (stability test). Specifically, these infants maintained transcutaneous peripheral oxygen saturation (SpO_2_) of 90% to 95%; partial pressure of carbon dioxide of 60 mm Hg or less; fraction of inspired oxygen (FIO_2_) less than 40%; Silverman-Andersen score^18^ of 6 or lower (score range: 0-10, with the highest score indicating extremely severe distress); and Apnea-Hypopnea Index of 2 events per hour or fewer, with continuous positive airway pressure (CPAP) of 7 cm H_2_O or less for NCPAP and flow of 7 L per minute or less for HHHFNC. This stability ensured safety of treatment and reliability of results, excluding the more compromised infants who needed mechanical ventilation for 5 days of life. Infants were randomized to 1 of the 2 NRS groups by block randomization. Software was designed to automatically generate a randomization code and to establish, in each research unit, a balance between patients with less than 28 weeks’ gestation and patients with at least 28 weeks’ gestation in both groups.

Nutritional data were recorded daily until the full enteral feeding (FEF) was reached. Respiratory data were recorded at each change of respiratory support until its discontinuation. The occurrence of comorbidity was recorded until discharge. Data were collected anonymously by a web-based database.

Each participating NICU adopted its own protocols for the clinical management of patients enrolled in the study, although respecting some standard criteria for enteral nutrition and NRS. The advancement of feeding was up to clinicians, but the upper limit was set at 30 mL/kg per day. The interruption of feeding was defined by signs of feeding intolerance from pathological abdominal physical examination (distention, discoloring, visible bowel loops, or pain), pathological gastric residual volume measurement (>100% of previous feed; bilious, hematic, or fecaloid), vomits and/or regurgitations, abnormal feces (mucous or hematic), and associated cardiorespiratory events. These signs were rated according to their severity as minor or major criteria whose combination guided the interruption of feeding (eTables 1, 2, and 3 in Supplement 2), as stated in the study protocol (Supplement 1).

Infants were bolus fed by nasogastric or orogastric tube; breastfeeding or bottle feeding was started according to the infant’s capability. The initial suggested setup for NRS was CPAP of between 5 and 7 cm H_2_O for NCPAP and flow of between 4 and 7 L per minute for HHHFNC. Failure and weaning criteria were adopted from current guidelines and consensus statement (eTable 3 in Supplement 2).^19,20,21^

The primary outcome was the time to reach FEF, defined as enteral intake of 150 mL/kg per day. Secondary nutritional outcomes were median daily enteral increment, at least 1 episode of feeding interruption, at least 1 episode of feeding interruption lasting more than 24 hours, pathological gastric residual volume, frequent vomits and/or regurgitations (≥3 in a day), abdominal distention score of 2 or higher (eTable 2 in Supplement 2), time to reach full oral feeding, and growth. Secondary respiratory outcomes were the number of days that the assigned NRS was maintained, number of patients who required more invasive respiratory support (mechanical ventilation or noninvasive intermittent positive-pressure ventilation), number of patients who changed group, number of patients who were weaned from respiratory support, and SpO_2_–FIO_2_ ratio at enrollment and at the first change of respiratory support.

Comorbidity occurred after randomization as NEC (Bell stage II or III), bronchopulmonary dysplasia (oxygen requirement at 36 weeks’ postmenstrual age or 28 days of life), pneumothorax, intraventricular hemorrhage (severe if higher than grade II),^22^ sepsis, patent ductus arteriosus requiring treatment, and retinopathy of prematurity. Length of hospital stay and mortality were also evaluated as secondary outcomes.

### Statistical Analysis

Statistical analysis was performed according to the intention-to-treat approach, considering all enrolled patients until discharge. The time to reach FEF was computed as number of days from randomization to FEF achievement and was estimated with the Kaplan-Meier method. Infants who were transferred or died before reaching FEF or whose parents had revoked consent were censored. Secondary nutritional outcomes, growth, respiratory outcomes, comorbidity, and length of hospital stay were analyzed using appropriate models and tests (eTable 4 in Supplement 2). Bonferroni correction was used to adjust for multiple comparisons, and significance level was set at *P* = .003. SAS, version 9.4 (SAS Institute Inc), was used to process data and fit statistical models.

A sample size of 123 infants per group was estimated to achieve 90% power to detect a difference of 30% on the primary outcome of time to reach FEF (which was projected to be 19.6 days based on data from the participating NICUs the year before the trial started) using a 2-sided log-rank test with a significance level of *P* = .05. Assuming a dropout of 13%, a total of 282 eligible infants were required.

## Results

**Figure 1.**
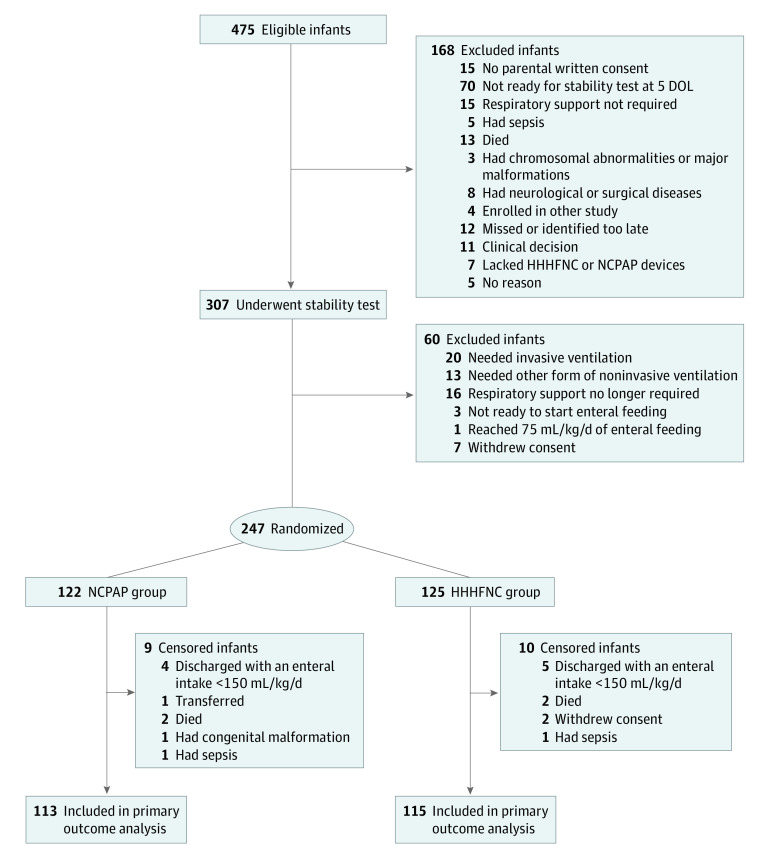
Study Design and Participant Distribution DOL indicates days of life; HHHFNC, heated humidified high-flow nasal cannula; NCPAP, nasal continuous positive airway pressure.

From the 13 participating NICUs, 475 infants were eligible for inclusion in the trial. Among them 168 infants (35.4%) were not able to undergo the stability test within day 5 of life and were excluded. The stability test was performed in 307 infants, 247 (80.5%) of whom passed the test and were randomized to the NCPAP arm (n = 122) or the HHHFNC arm (n = 125) (Figure 1). These infants had a median (IQR) gestational age of 28 (27-29) weeks and included 130 girls (52.6%) and 117 boys (47.4%).

Nineteen infants were censored, and their characteristics are described in eTable 5 in Supplement 2. Infants’ distributions by NICU and NRS group and by NICU and gestational age are provided in eFigures 1 and 2 in Supplement 2. At enrollment, the 2 groups were similar in days of life when enteral feeding was started, amount of enteral feeding, frequency of being fed exclusively with human milk (mother or donor milk), as well as FIO_2_, SpO_2_, and SpO_2_–FIO_2_ ratio. Baseline characteristics of the study population by group are provided in Table 1. The number of infants who were fed with fortified human milk and the volume of feed at which fortification started were similar in the NCPAP and HHHFNC groups.

**Table 1.** Characteristics of the Study Population by Arm

Baseline characteristic	Group, No. (%)	
	NCPAP	HHHFNC
All participants, No.	122	125
Steroid prophylaxis	112 (91.8)	115 (92.0)
Maternal diabetes	18 (14.8)	14 (11.2)
Maternal hypertension	23 (18.9)	15 (12.0)
Multiple pregnancy	33 (27.0)	38 (30.4)
Cesarean delivery	77 (63.1)	88 (70.4)
Female sex	62 (50.8)	68 (54.4)
Male sex	60 (49.2)	57 (45.6)
GA <28 wk	52 (42.6)	56 (44.8)
Birth weight, mean (SD), g	1054 (220)	1085 (243)
GA, median (IQR), wk	28 (27-29)	28 (27-29)
Birth weight, mean (SD), *z* score^a^	0.20 (0.88)	0.32 (0.88)
SGA^b^	9 (7.4)	7 (5.6)
LGA^c^	11 (9.0)	15 (12.0)
IUGR	20 (16.4)	18 (14.4)
Apgar score at 5 min, median (IQR)	8 (7-8)	8 (7-8)
Age at recruitment, median (IQR), d	4 (3-6)	5 (3-6)
Nutritional characteristics		
Start enteral feeding, median (IQR), d	0 (0-1)	0 (0-1)
Enteral intake at recruitment, median (IQR), mL/kg/d	23 (15-43)	25 (15-45)
Exclusively fed with human milk	97 (79.5)	100 (80.0)
Respiratory characteristics		
Surfactant therapy	64 (52.5)	71 (56.8)
NRS before recruitment: NCPAP	112 (91.8)	112 (89.6)
FIO_2_ at recruitment, median (IQR), %	21 (21-25)	21 (21-25)
SpO_2_ at recruitment, median (IQR), %	97 (95-98)	97 (96-98)
SpO_2_–FIO_2_ ratio at recruitment, median (IQR)	4.6 (4.2-4.7)	4.6 (3.9-4.7)

Abbreviations: FIO_2_, fraction of inspired oxygen; GA, gestational age; HHHFNC, heated humidified high-flow nasal cannula; INeS, Italian Neonatal Study; IUGR, intrauterine growth retardation; LGA, large for gestational age; NCPAP, nasal continuous positive airway pressure; NRS, noninvasive respiratory support; SGA small for gestational age; SpO_2_, peripheral oxygen saturation.

^a^ The *z* score was calculated using the INeS charts.^23^

^b^ SGA was defined as birth weight less than the 10th percentile in the INeS charts.^23^

^c^ LGA was defined as birth weight greater than the 90th percentile in the INeS charts.^23^

### Primary Outcome

**Figure 2.**
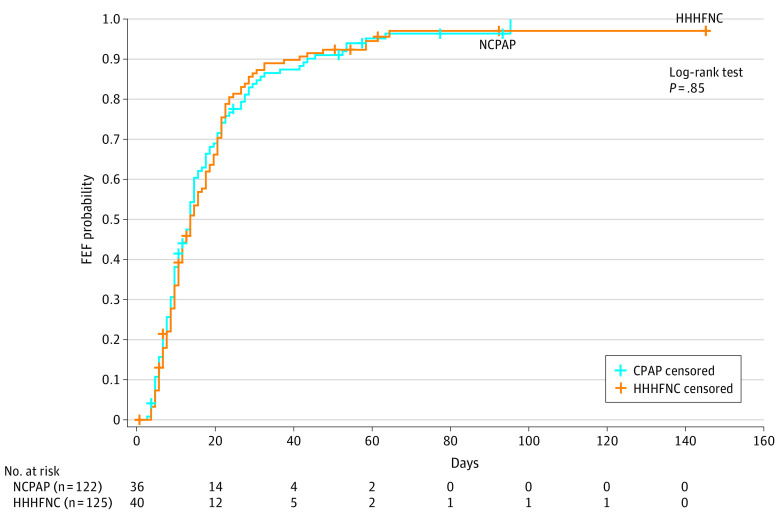
Time to Reach Full Enteral Feeding (FEF) in the Nasal Continuous Positive Airway Pressure (NCPAP) and Heated Humidified High-Flow Nasal Cannula (HHHFNC) Arms

The estimates of FEF probability showed no differences between the 2 groups (Figure 2). The median time to reach FEF was 14 days (95% CI, 11-15 days) in the NCPAP group and 14 days (95% CI, 12-18 days) in the HHHFNC group (log-rank test: *P* = .85). Stratified analysis by gestational age did not show differences between the 2 groups (eFigure 3 in Supplement 2).

### Secondary Outcomes

There were no differences between the 2 groups in the risk of having at least 1 episode of feeding interruption, at least 1 episode of feeding interruption lasting more than 24 hours, at least 1 episode of pathological gastric residual volume, at least 3 episodes of vomiting or regurgitation in a day, an abdominal distension score of 2 or higher, and at least 1 cardiorespiratory event.^21^ Risk and relative risk of secondary nutritional outcomes are reported in Table 2. No differences in the mean daily increment were observed between the NCPAP vs HHHFNC arms (10.8 [95% CI, 9.6-12.2] mL/kg/d vs 11.1 [95% CI, 9.8-12.5] mL/kg/d). The mean time to reach full oral feeding was similar in both NCPAP and HHHFNC groups (49 [95% CI, 42-57] days vs 48 [95% CI, 41-57] days).

**Table 2.** Risk and Relative Risk of Nutritional Outcomes^a^

Nutritional outcome	Risk (95% CI)		HHHFNC vs NCPAP, RR (95% CI)
	NCPAP group	HHHFNC group	
≥1 Feeding interruption	0.41 (0.33-0.51)	0.47 (0.39-0.57)	1.15 (0.87-1.53)
≥1 Feeding interruption lasting >24 h	0.21 (0.15-0.30)	0.26 (0.20-0.35)	1.24 (0.79-1.94)
Abdominal distension score^b^	0.35 (0.28-0.45)	0.43 (0.35-0.53)	1.23 (0.90-1.68)
≥3 Regurgitations or vomits per d	0.12 (0.08-0.20)	0.08 (0.04-0.15)	0.65 (0.30-1.39)
≥1 Pathological GRV	0.68 (0.60-0.77)	0.77 (0.70-0.84)	1.13 (0.97-1.32)
≥3 Cardiorespiratory events^c^	0.66 (0.58-0.75)	0.61 (0.53-0.70)	0.93 (0.77-1.13)

Abbreviations: GRV, gastric residual volume; HHHFNC, heated humidified high-flow nasal cannula; NCPAP, nasal continuous positive airway pressure; RR, relative risk.

^a^ The estimates were computed using Poisson regression with robust error variance.

^b^ Abdominal distention score of 2 or higher, with higher scores indicating greater severity.^21^

^c^ Episodes of desaturation with blood oxygen saturation below 80%, episodes of bradycardia with heart rate below 80 beats per minute, or episodes of apnea lasting more than 20 seconds or more than 5 seconds if followed by desaturation or bradycardia.

### Growth and Respiratory Outcomes

Weight growth between randomization and time to reach FEF was similar in the 2 groups, with a mean difference of 0.81 g/kg per day (95% CI, −1.50 to 3.12 g/kg/d). The mean number of days that the assigned NRS was maintained was shorter in the NCPAP group than in the HHHFNC group (8.9 [95% CI, 6.8-11.8] days vs 12.1 [95% CI, 9.6-15.2] days). Between randomization and time to reach FEF, 83 infants (68.0%) in the NCPAP group and 76 infants (60.8%) in the HHHFNC group changed or discontinued their assigned NRS. Specifically, at the first change of NRS, 14 infants (16.9%) who were treated with NCPAP vs 18 infants (23.7%) who were treated with HHHFNC required more invasive respiratory support (ie, mechanical ventilation or noninvasive intermittent positive-pressure ventilation). This change was needed later at a mean of 7.3 days (95% CI, 3.0-17.0 days) in the NCPAP group compared with 3.7 days (95% CI, 1.5-8.7 days) in the HHHFNC group after randomization. Forty-eight infants (57.8%) in the NCPAP group vs 35 infants (46.1%) in the HHHFNC group had improvement and discontinued any respiratory support at a mean of 4.9 days (95% CI, 3.5-6.9 days) and 9.1 days (95% CI, 6.7-12.4 days), respectively, after randomization (*P* < .001). Furthermore, 21 infants (25.3%) who were treated with NCPAP vs 23 infants (30.3%) who were treated with HHHFNC switched NRS groups at a mean of 4.6 days (95% CI, 1.3-16.2 days) and 3.0 days (95% CI, 0.9-10.7 days), respectively, after randomization.

Forty-four infants changed NRS groups. The number of patients who switched due to treatment ineffectiveness was lower in the NCPAP group compared with the HHHFNC group (1 [4.8%] vs 17 [73.9%]; *P* < .001). Conversely, the number of infants who switched due to their inability to tolerate the respiratory interface was higher in the NCPAP group compared with the HHHFNC group (8 [38.1%] vs 0 [0%]; *P* = .001]. At the time of the change, the median (IQR) SpO_2_–FIO_2_ ratio was higher in the NCPAP group than the HHHFNC group (4.6 [4.1-4.7] vs 3.7 [3.2-4.0]; *P* < .001).

### Comorbidity Outcomes, Length of Hospital Stay, and Mortality

No differences were observed between the 2 groups in the frequency of bronchopulmonary dysplasia, retinopathy of prematurity, sepsis, and patent ductus arteriosus (Table 3). As expected, there were only a few cases of NEC (4 in the NCPAP group; 3 in the HHHFNC group), pneumothorax (2 in the NCPAP group; 0 in the HHHFNC group), severe intraventricular hemorrhage (0 in the NCPAP group; 2 in the HHHFNC group), and death before FEF achievement (2 in the NCPAP group; 2 in the HHHFNC group); thus, a comparison between groups could not be performed. The mean length of hospital stay was similar in both groups (63.9 [95% CI, 58.6-69.7] days vs 63.5 [95% CI, 58.3-69.1] days).

**Table 3.** Risk and Relative Risk of Comorbidity Outcome^a^

Comorbidity outcome	Risk (95% CI)		HHHFNC vs NCPAP, RR (95% CI)
	NCPAP group	HHHFNC group	
BPD	0.17 (0.11-0.25)	0.18 (0.12-0.27)	1.06 (0.61-1.84)
ROP	0.13 (0.09-0.21)	0.15 (0.10-0.23)	1.14 (0.62-2.11)
Sepsis	0.32 (0.24-0.41)	0.41 (0.33-0.51)	1.30 (0.92-1.82)
PDA	0.15 (0.09-0.24)	0.14 (0.08-0.24)	0.95 (0.46-1.93)

Abbreviations: BPD, bronchopulmonary dysplasia; HHHFNC, heated humidified high-flow nasal cannula; NCPAP, nasal continuous positive airway pressure; PDA, patent ductus arteriosus; ROP, retinopathy of prematurity; RR, relative risk.

^a^ The estimates were computed using Poisson regression with robust error variance.

## Discussion

To our knowledge, this multicenter randomized clinical trial was the first to investigate the effects on feeding intolerance of the 2 most common techniques of NRS (NCPAP and HHHFNC) in preterm infants at high risk for feeding problems. We found no differences in time to reach FEF between the NCPAP and HHHFNC groups, and the population was not analyzed by different gestational age classes.

These findings disproved our hypothesis that HHHFNC might affect feeding intolerance to a lesser extent than NCPAP, but they are in agreement with the results reported by Amendolia et al,^5^ who studied 104 very-low-birth-weight infants and did not find differences in time to reach FEF between infants who were treated with NCPAP vs those who used HHHFNC. Conversely, a single-center trial in 94 extremely-low-birth-weight infants found that HHHFNC reduced the time to reach FEF and NEC incidence compared with NCPAP.^15^ However, that study is not comparable with the present trial since the occurrence of NEC was 5- to 10-fold higher and the antenatal corticosteroids rate was 4- to 5-fold lower.^15^ A recent meta-analysis supports this hypothesis, reporting that earlier FEF was reached with HHHFNC compared with NCPAP as the primary respiratory support.^16^ However, the authors emphasized the high heterogeneity of the results,^16^ suggesting that results might be affected by the lack of protocols and other undetected bias.

We based the study hypothesis on the premise that infants with HHHFNC rather than NCPAP treatment experience less feeding intolerance that is secondary to less gastric distension from high positive pressure, discomfort from nasal prongs and nasopharyngeal tube, stress and fatigue from overhandling, and hesitation in increasing volume of enteral feeding. The results of this trial disproved this hypothesis. To explain this disagreement, we can argue that the high flow of oxygen or air delivered by HHHFNC devices increases the pharyngeal pressure by 0.8 cm H_2_O for each 1 L per minute^24^; therefore, HHHFNC can have the same complications as NCPAP on gastric and abdominal distension. In both NRS groups, the high frequency of human milk–exclusive feeding, which favored the tolerance of enteral nutrition, might counteract the discomfort and stress from NRS. Moreover, the exact definition of feeding intolerance and standardization of the management of feeding increase or interruption helped with limiting the delays in the advancement of enteral feeding, shortening the time to reach FEF in the present population compared with the previous studies.^5,15^

Concerning the respiratory outcomes, NCPAP and HHHFNC had similar rates of failure and change in NRS. However, the change from NCPAP to HHHFNC was mainly due to poor compliance with the interface, and HHHFNC was used as a weaning technique from NCPAP. The switch from HHHFNC to NCPAP was mainly due to lower respiratory efficacy of HHHFNC. The SpO_2_–FIO_2_ ratio at the time the NRS was changed was higher in infants who were being treated with NCPAP than in infants using HHHFNC, which is consistent with findings of a previous study that demonstrated a shorter time to wean from NRS when patients were treated with NCPAP compared with HHHFNC.^25^

### Limitations

A major limitation of this study was the inability to mask clinicians to the randomized treatment, which may have influenced the decision to change the assigned NRS. On the other hand, the presence of well-defined rules for advancement and interruption of feeding as well as for weaning and failure of NRS, and the blinded analysis by biostatisticians from outside the clinical teams ensured the reliability of results. Another limitation was that the study focused on a population of stable infants; thus, the efficacy of the 2 techniques cannot be generalized to their use as a first-intention treatment. However, we designed a pragmatic study in which clinicians could modify or discontinue the respiratory support and enteral nutrition, providing results that are useful in everyday clinical practice.

## Conclusions

The results of this trial showed that there was no difference in feeding intolerance between infants in the NCPAP and HHHFNC groups, although some short-term respiratory outcomes were better with NCPAP. These findings suggest that clinicians should tailor respiratory care by choosing and switching between the 2 NRS techniques on the basis of their respiratory efficacy and patient compliance, regardless of the possible effects on feeding intolerance.

## Notes

Supplement 1.
Trial Protocol

Supplement 2. **eTable 1.** Criteria for the Interruption of Enteral Feeding **eTable 2.** Abdominal Distension Score **eTable 3.** Minimal Standard Criteria for Respiratory Support **eTable 4.** Secondary Outcomes Statistical Analysis **eTable 5.** Characteristics of Censored Infants **eFigure 1.** Infants Distribution by Centre and Arm **eFigure 2.** Infants Distribution by Centre and GA **eFigure 3.** Time to Reach Full Enteral Feeding (150 mL/kg/day) in NCPAP and HHHFNC Arms by GA

Supplement 3.
Nonauthor Collaborators

Supplement 4.
Data Sharing Statement
