# Supplementary material for: Effect of Nasal Continuous Positive Airway Pressure vs Heated Humidified High-Flow Nasal Cannula on Feeding Intolerance in Preterm Infants With Respiratory Distress Syndrome: The ENTARES Randomized Clinical Trial
Source: JAMA Netw Open. 2023 Jul 12;6(7):e2323052. doi: 10.1001/jamanetworkopen.2023.23052 (PMC10339152; doi:10.1001/jamanetworkopen.2023.23052)
Supplement: Supplement 1. — Trial Protocol [file jamanetwopen-e2323052-s001.pdf]

Supplement 1. Study protocol and statistical analysis plan.

This supplement contains the following items:

- Original protocol (Italian), final protocol, summary of changes.
- Original statistical analysis plan, final statistical analysis plan, summary of changes.

Summary

- Original protocol (Italian) 2
- Final protocol 10
- Summary of changes (protocol) 20
- Original statistical analysis plan 21
- final statistical analysis plan 22
- Summary of changes (analysis plan) 24

Original protocol (Italian)

INTRODUZIONE

Premessa

Il distress respiratorio e la broncodisplasia rappresentano uno dei maggiori problemi nel neonato pretermine. Offrire un adeguato supporto respiratorio limitando il danno polmonare è l'obiettivo principale del neonatologo. Le tecniche di supporto respiratorio non-invasive, associate all'uso del surfactante, permettono di evitare o limitare l'uso della ventilazione meccanica convenzionale e sono associate a migliori outcomes in termini di mortalità e di complicanze short- e long-term, tra cui la displasia broncopolmonare.[1,2]

Le tecniche di supporto respiratorio non invasivo attualmente più diffuse nelle Unità di Terapia Intensiva Neonatale sono la NCPAP e gli HHHFNC. Le evidenze scientifiche al momento disponibili indicano che le due tecniche permettono di ottenere risultati comparabili in termini di efficacia e rischio di broncodisplasia.[3,4] Ancora poco studiato, è il loro impatto sulle problematiche nutrizionali del pretermine, in particolare sulla tolleranza alimentare e sul rischio di NEC.

La nostra ipotesi è che le tecniche di supporto respiratorio non invasivo possano avere differente impatto sulla tolleranza dell'alimentazione enterale nel neonato pretermine.

Scopo dello studio ed impatto sulla pratica clinica

Valutare gli effetti di NCPAP e HHHFNC sulla tolleranza alimentare nel neonato pretermine.

L'individuazione della tecnica di supporto respiratorio più adatta per il pretermine con intolleranza alimentare potrebbe ridurre le complicanze gastrointestinali, migliorare la crescita e ridurre i tempi di ospedalizzazione.

Disegno dello studio

Lo studio prevede un trial randomizzato controllato multicentrico su neonati pretermine sottoposti a diverse modalità di supporto respiratorio (NCPAP o HHHFNC), nel quale verranno confrontati il tempo di raggiungimento della full enteral feeding (FEF) e alcuni parametri di valutazione della tolleranza alimentare. Il disegno dello studio è schematizzato in figura 1.

Setting dello studio

Lo studio si svolgerà all'interno della Terapia Intensiva Neonatale – S.C. Neonatologia U. – Ospedale Sant'Anna – Città della Salute e della Scienza di Torino che costituirà il centro coordinatore dello studio.

Intervento proposto

In base alla randomizzazione i pazienti eligibili saranno assegnati ad uno dei due bracci previsti (NCPAP o HHHFNC) e trattati secondo i protocolli abitualmente utilizzati in ogni centro partecipante seppur nel rispetto di alcuni criteri standard di ventilazione e di alimentazione che verranno definiti nei paragrafi seguenti. La partecipazione allo studio non comporterà l'esecuzione di esami di laboratorio o strumentali aggiuntivi.

PAZIENTI E METODI

Criteri di inclusione

Verranno considerati arruolabili tutti i neonati con età ≤ 7 giorni ed età gestazionale compresa tra 25 settimane + 0 giorni e 29 settimane + 6 giorni che entro i primi 5 giorni di vita siano stati assistiti mediante supporto respiratorio non invasivo (NCPAP o HHHFNC) e che si siano dimostrati stabili,

nella modalità di supporto respiratorio scelto dai clinici, per almeno 48 ore.
I criteri per definire la stabilità nella modalità di supporto respiratorio prescelto sono elencati di seguito[5,6]:

- $SatO_{2\ TC}$ 90-95%
- $pCO_2 \leq 60$ mmHg
- $FiO_2 < 40\%$
- Silverman score ≤ 6
- Episodi di apnea ≤ 2 episodi/ora

in presenza di CPAP ≤ 7 cmH_2O (NCPAP) o flusso ≤ 6 L/min (HHHFNC)
Inoltre, saranno considerati arruolabili solo i neonati che, al momento della randomizzazione, abbiano già iniziato l'alimentazione enterale o siano ritenuti idonei ad iniziarla.
I neonati potranno partecipare allo studio solo previo ottenimento del consenso informato da parte dei genitori.

Criteri di esclusione
Saranno esclusi dallo studio i neonati che, al momento dell'arruolamento, saranno affetti da patologie neurologiche, malformative, chirurgiche o sepsi.

Randomizzazione
I neonati arruolati saranno assegnati ad uno dei due bracci (NCPAP o HHHFNC) tramite una randomizzazione a blocchi, studiata per ottenere in ciascun centro un equilibrio tra i due bracci sia per i pazienti con età gestazionale < di 28 settimane che per quelli con età gestazionale ≤ 28. La randomizzazione ed il conseguente arruolamento saranno possibili dopo aver dimostrato la stabilità del paziente in una delle due modalità di supporto respiratorio (NCPAP o HHHHFNC), secondo i criteri precedentemente descritti.

Ventilazione
All'arruolamento l'impostazione dei parametri di ventilazione dovrà essere tale da consentire adeguate saturazioni transcutanee di ossigeno (90-95%) ed individuata a partire dai seguenti setup iniziali.

Setup iniziale suggerito (NCPAP/HHHFNC)

	NCPAP [6]	***HHHFNC*** [5]
CPAP (cm H_2O)	5 – 7	-
Flusso (L/min)	-	4 – 6
FiO_2 %	Tale da ottenere pO_2 = 50 – 60 mmHg** e $SatO_{2\ TC}$ = 90 – 95%	

** Valori ottenuti da EGA capillari

I criteri per il tentativo di svezzamento ed il fallimento del supporto respiratorio non invasivo sono riportati di seguito:

Criteri per tentare lo svezzamento

	NCPAP	***HHHFNC***
CPAP (cm H_2O)	< 4	-

Flusso (L/min)	-	< 2
FiO_2 %	< 25% in presenza di pO_2 = 50 – 60 mmHg** e $SatO_{2\,TC}$ = 90 – 95%	

** Valori ottenuti da EGA capillari

<u>Criteri di fallimento</u>

	NCPAP/HHHFNC
FiO_2 %	> 40
*pH**	< 7.2
*pCO_2 (mmHg)**	> 65
Desaturazioni ($SatO_{2\,TC}$ ≤ 80%)	≥ 3 episodi/ora
*Apnea (> 20 s) / bradicardia (FC ≤ 80 bpm)***	≥ 3 episodi/ora
Silverman score	> 6

* Valori ottenuti da EGA capillari
** Pazienti in terapia con caffeina

Alimentazione

Tutti i neonati arruolati dovranno iniziare l’alimentazione enterale, se non iniziata precedentemente, al momento della randomizzazione.

I neonati potranno essere alimentati secondo i protocolli presenti nei singoli centri partecipanti allo studio. Tuttavia, i centri partecipanti dovranno attenersi ai seguenti criteri circa l’incremento dell’alimentazione enterale e la sospensione dei pasti.

<u>Criteri di incremento dell’alimentazione enterale</u>

La decisione di incrementare i pasti è libera e basata sulle condizioni cliniche, gli incrementi giornalieri potranno essere personalizzati fino ad un incremento massimo di 30 mL/kg/die.

<u>Criteri di sospensione del pasto:</u>

La decisione di sospendere il pasto dovrà attenersi ai criteri riportati in tabella 1.

Monitoraggio

Lo studio sarà condotto secondo il modello dell’intention-to-treat. Tutti i neonati arruolati saranno monitorati sino al momento della dimissione, qualsiasi siano gli eventi clinici intercorrenti, compreso il fallimento della modalità di supporto respiratorio assegnata all’arruolamento.

A seguito dell’arruolamento un paziente sarà escluso dallo studio solo in caso di decesso o trasferimento presso altra Struttura prima del raggiungimento della full enteral feeding.

Per tutti i neonati arruolati verranno annotati parametri di ventilazione/respirazione, clinici e di alimentazione.

<u>Parametri di ventilazione/respirazione:</u>

- Modalità di supporto respiratorio in uso (NCPAP, HHHFNC, BiPAP, (s)NIPPV, HFVO, VM)
- PEEP/CPAP (cmH_2O)
- PIP (cmH_2O)
- Flusso (L/min)
- FiO_2 (%)

Parametri clinici

- Frequenza respiratoria (atti/min)
- Saturazione % di $O_{2\ TC}$
- EGA capillare
- Episodi di apnea (> 20 s)/die
- Episodi di bradicardia (FC ≤ 80 bpm)/die
- Episodi di desaturazione ($SatO_{2\ TC}$ ≤ 80%)/die
- Silverman score

Tutti i parametri di ventilazione/respirazione e clinici saranno annotati all'arruolamento, in occasione dei cambi di modalità di assistenza respiratoria e in occasione dell'esecuzione dell'EGA capillare, eccezion fatta per il monitoraggio degli episodi di apnea che sarà quotidiano e sino che sarà necessari assistenza respiratoria.

Parametri di alimentazione:

- Apporto di nutrizione parenterale (mL/kg/die)
- Apporto di alimentazione enterale (mL/kg/die)
- Apporto calorico totale (Kcal/kg/die)
- Tipo di alimento (LM, LD, latte formulato)
- Modalità di alimentazione (enterale continua, bolo, gavage, suzione)
- Eventuale fortificazione e relative modalità (tipo di fortificante, fortificazione standard, fortificazione personalizzata)
- n. sospensioni del pasto (eventi/die) (tabella 1)
- n. pasti non somministrati (eventi/die) (tabella 2)
- n. ristagni patologici* (eventi/die) (tabella 2)
- n. vomiti e/o rigurgiti (eventi/die)[7–10]
- Grado di distensione addominale (score medio/die) (tabella 2)

* *la decisione di valutare i ristagni gastrici sarà prerogativa di ogni centro. Ai fini dello studio saranno considerati patologici i ristagni gastrici descritti tra i criteri minori e maggiori elencati in tabella 1.*

All'arruolamento (T_0), al raggiungimento della full enteral feeding (FEF) (T_1) e alla dimissione (T_2) verranno annotate le seguenti variabili antropometriche: peso (g), lunghezza (cm) e circonferenza cranica (cm).

Outcome primario:

- Tempo di raggiungimento della FEF, definita come quoziente idrico di 150 mL/kg/die assunto per via enterale.

Outcomes secondari:

- tempo di FEF/2 (n. giorni)
- n. sospensioni del pasto (eventi/die), valutato fine ventilazione, FEF/2, FEF
- n. pasti non somministrati (eventi/die), valutato a fine ventilazione, FEF/2, FEF
- n. ristagni patologici (eventi/die), valutato a fine ventilazione, FEF/2, FEF
- n. vomiti e/o rigurgiti (eventi/die), valutato a fine ventilazione, FEF/2, FEF
- distensione addominale (punteggio medio), valutato a fine ventilazione, FEF/2, FEF
- accrescimento ponderale (Δ Z-score), valutato a fine ventilazione, FEF/2, FEF
- durata del supporto respiratorio assegnato alla randomizzazione (n. giorni)

- durata totale della necessità di supporto respiratorio/ventilatorio (n. giorni)
- fallimento della modalità di supporto respiratorio assegnata alla randomizzazione (si/no)
- durata ricovero (n. giorni)
- complicazioni (NEC, perforazione intestinale, PNX, BPD, ROP)

Analisi statistica

I dati saranno analizzati secondo il modello intention-to-treat.

L'outcome primario sarà valutato mediante un'analisi della sopravvivenza con distribuzione non parametrica. Gli outcomes secondari saranno valutati mediante test esatto di Fisher o modelli lineari generalizzati appropriati.

Considerando un rapporto tra i soggetti dei due gruppi di 1:1, un valore di α=0.05 e β=.10 è stato calcolato che per osservare una differenza del 30% tra i 2 gruppi siano necessari 123 pazienti per braccio, basandosi sui dati osservati in un gruppo sequenziale di pazienti < 29 settimane di età gestazionale ricoverati presso le Terapie Intensive Neonatali dei centri partecipanti allo studio da gennaio a giugno 2017 (tempo medio di FEF: 19,6 giorni, delta: 5.7). Ipotizzando una frequenza di drop-out del 13%, si prevede di arruolare 282 neonati eligibili.

Figura 1. Disegno dello studio

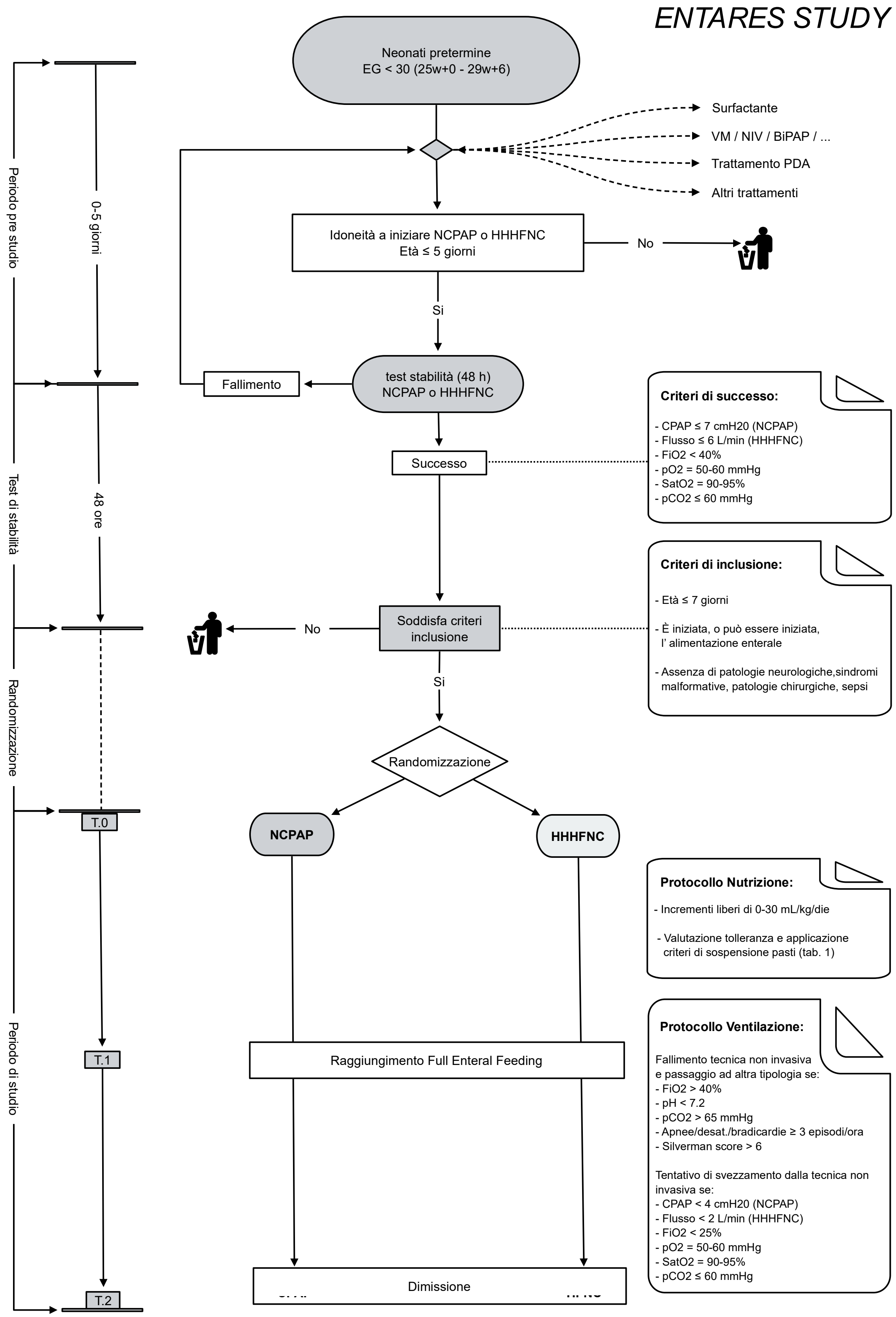

Tabella 1. Criteri di intolleranza alimentare

	Criteri Minori	*Criteri Maggiori*
Esame Obiettivo	• Addome globoso<br>• Anse intestinali visibili<br>• Addome teso responsivo alla detensione gastrica/ sondaggio	• Discromia cutanea addominale<br>• Addome teso non responsivo alla detensione gastrica/ sondaggio<br>• Addome pastoso/dolente
Rigurgiti/vomiti	• ≤ 2 episodi in 1 periodo interprandiale o nelle 3 ore precedenti (se non alimentato)	• > 2 episodi nel periodo interprandiale o nelle 3 ore precedenti (se non alimentato)<br>• Vomito biliare/ematico
Ristagni gastrici	• Ristagno < 100% (biliari o con frustoli ematici)	• Ristagno ematico/fecaloide<br>• Ristagno ≥ 100% del pasto precedente
Alvo	• Feci mucose	• Feci ematiche

**0-1 criteri minori**:	alimentazione con incrementi secondo protocollo
**2 criteri minori**:	proseguire alimentazione invariata, ripetere la valutazione prima del pasto successivo e valutare i ristagni gastrici se non precedentemente effettuato;<br>se presenti 2 criteri minori in almeno 2 valutazioni consecutive considerare la riduzione del pasto
**1 criterio maggiore o 3 criteri minori**:	sospendere l'alimentazione e ripetere la valutazione prima del pasto successivo

Tabella 2. Grado di distensione addominale

	Punteggio
Addome normoespanso	0
Addome globoso non teso	1
Addome globoso e teso responsivo alla detensione gastrica/sondaggio rettale	2
Addome globoso e teso non responsivo alla detensione gastrica/sondaggio rettale	3

BIBLIOGRAFIA

1. Aly H. Ventilation without tracheal intubation. *Pediatrics*. 2009;124(2):786-789. doi:10.1542/peds.2009-0256.

2. Ramanathan R, Sekar KC, Rasmussen M, Bhatia J, Soll RF. Nasal intermittent positive pressure ventilation after surfactant treatment for respiratory distress syndrome in preterm infants <30 weeks' gestation: a randomized, controlled trial. *J Perinatol Off J Calif Perinat Assoc*. 2012;32(5):336-343. doi:10.1038/jp.2012.1.

3. Wilkinson D, Andersen C, O'Donnell CP, De Paoli AG, Manley BJ. High flow nasal cannula for respiratory support in preterm infants. 2016. doi:10.1002/14651858.CD006405.pub3.

4. Kotecha SJ, Adappa R, Gupta N, Watkins WJ, Kotecha S, Chakraborty M. Safety and Efficacy of High-Flow Nasal Cannula Therapy in Preterm Infants: A Meta-analysis. *PEDIATRICS*. 2015;136(3):542-553. doi:10.1542/peds.2015-0738.

5. Yoder BA, Manley B, Collins C, et al. Consensus approach to nasal high-flow therapy in neonates. *J Perinatol Off J Calif Perinat Assoc*. March 2017. doi:10.1038/jp.2017.24.

6. Salvo V, Lista G, Lupo E, et al. Comparison of three non-invasive ventilation strategies (NSIPPV/BiPAP/NCPAP) for RDS in VLBW infants. *J Matern-Fetal Neonatal Med Off J Eur Assoc Perinat Med Fed Asia Ocean Perinat Soc Int Soc Perinat Obstet*. July 2017:1-22. doi:10.1080/14767058.2017.1357693.

7. Mihatsch WA, Pohlandt F, Franz AR, Flock F. Early feeding advancement in very low-birth-weight infants with intrauterine growth retardation and increased umbilical artery resistance. *J Pediatr Gastroenterol Nutr*. 2002;35(2):144-148.

8. Parker L, Torrazza RM, Li Y, Talaga E, Shuster J, Neu J. Aspiration and Evaluation of Gastric Residuals in the Neonatal Intensive Care Unit: State of the Science. *J Perinat Neonatal Nurs*. 2015;29(1):51-59. doi:10.1097/JPN.0000000000000080.

9. Li Y-F, Lin H-C, Torrazza RM, Parker L, Talaga E, Neu J. Gastric Residual Evaluation in Preterm Neonates: A Useful Monitoring Technique or a Hindrance? *Pediatr Neonatol*. 2014;55(5):335-340. doi:10.1016/j.pedneo.2014.02.008.

10. Gephart SM, Fleiner M, Kijewski A. The ConNECtion Between Abdominal Signs and Necrotizing Enterocolitis in Infants 501 to 1500 g. *Adv Neonatal Care Off J Natl Assoc Neonatal Nurses*. 2017;17(1):53-64. doi:10.1097/ANC.0000000000000345.

Final protocol

Background
Respiratory distress syndrome (RDS) and feeding intolerance are common conditions in preterm infants and among the major causes of neonatal mortality and morbidity.
For many years, preterm infants with RDS have been treated with mechanical ventilation increasing risks of acute lung injury and bronchopulmonary dysplasia.
In recent years non-invasive ventilation techniques have been developed. Showing similar efficacy and risk of bronchopulmonary dysplasia, nasal continuous positive airway pressure (NCPAP) and heated humidified high flow nasal cannula (HHHFNC) became the most widespread techniques in neonatal intensive care units (NICUs). However, their impact on nutrition, particularly on feeding tolerance and risk of complications, is still unknown in preterm infants.
Aim of the study is to evaluate the impact of NCPAP vs HHHFNC on enteral feeding and to identify the most suitable technique for preterm infants with RDS.

AIM
Aim of the study is to evaluate the effects of different type of non invasive respiratory support (NCPAP vs HHHFNC) on feeding tolerance in preterm infants with RDS

METHODS
Study design and setting
The study has been designed as a multicenter randomized no mask controlled trial; it will involve the major Italian neonatal intensive care units (NICUs) and will be coordinated by the NICU of the University of Turin.
Inclusion criteria
All infants admitted to the NICUs with a gestational age between 25 and 29 weeks and who will have met the following inclusion criteria will be consecutively enrolled into the study:

1. Presence of RDS;
2. Period of stability on HHHFNC or NCPAP for at least 48 hours in the first 5 days of life ($SatO_{2\,TC}$ 90-95%, $pCO_2 \leq 60$ mmHg, $FiO_2 < 40\%$, Silverman score[1] ≤ 6, ≤ 2 apnea episodes/hour with CPAP ≤ 7 cmH_2O if on NCPAP and flow ≤ 7 L/min if on HHHFNC);
3. ≤ 7 days of life;
4. Suitability to start enteral feeding (if already started it should be less than 75 mL/Kg/die);
5. Parental written consent.

Exclusion criteria

1. Neurological or surgical diseases;
2. Sepsis;
3. Chromosomal abnormalities;
4. Major malformations.

Recruitment and randomization
Informed written consent will be signed by both the parents and sufficient time will be allowed for consent. Non-Italian speaking parents will only be asked for their consent if an adult interpreter is available. Trust interpreter and link worker services will be used to support involvement of participants whose first language is not Italian.
Eligible patients will be allocated to one of the two arms (NCPAP or HHHFNC) by block randomization. A software has been designed to automatically generate a randomization code and

to obtain, in each research unit, a balance between patients with gestational age <28 weeks and ≥ 28 weeks in both arms. The randomization software will be available for all research units, on a password-protected platform into the ENTARES website and will generate a randomization sequence to which all clinicians are blind.

Monitoring and data collection

Each research unit will adopt its own protocols for clinical management of the patients enrolled into the study, although respecting some minimal standard criteria for respiratory support and enteral nutrition, common for all participating units and defined as follows.

<u>Minimal standard criteria for respiratory support</u>

Suggested initial setup:[2,3]

- CPAP between 5 and 7 cmH_2O if on NCPAP and flow between 4 and 7 L/min if on HHHFNC;
- FiO_2 is set as to reach pO_2 = 50 - 60 mmHg (capillary/arterial blood gas test) and $SatO_{2\ TC}$ = 90 - 95%.

Criteria to try weaning: [2,3]

- CPAP < 4 cmH_2O if on NCPAP and flow < 2 L/min if on HHHFNC
- FiO_2 < 25% to maintain pO_2 = 50 - 60 mmHg (capillary/arterial blood gas test) and $SatO_{2\ TC}$ = 90 - 95%.

Failure criteria: [2,3]

- FiO_2 > 40%
- pH < 7.2
- pCO_2 > 65 mmHg
- ≥ 3 episodes of desaturations ($SatO_{2\ TC}$ ≤ 80%) per hour
- ≥ 3 episodes of apnea (> 20 sec) and/or bradycardia (FC ≤ 80 bpm) per hour
- Silverman score[1] > 6

<u>Minimal standard criteria for enteral nutrition</u>

The decision to increase volume of feeds is up to clinicians and according to the protocol used in their own NICU, however, a maximum cut-off for feeding progression was set at 30 mL/Kg/day.[4,5]
The indications for the interruption of feeding are based on abdominal examination, on the occurrence of vomits/regurgitations, cardiorespiratory events and on the evaluation of alvus and gastric residual volumes (evaluated if required by the protocol in use) as detailed in Table 1.[6–8] A score system was developed to evaluate abdominal distension (Table 2).

Table 1. Criteria for the interruption of enteral feeding.

	Minor Criteria	*Major Criteria*
Physical examination	• Abdominal distension<br>• Visible bowel ansa<br>• Abdominal distension responsive to gastric detension/rectal stimulation	• Dyschromic abdominal wall<br>• Abdominal distension not responsive to gastric detension/rectal stimulation<br>• Painful abdomen
Regurgitations/vomits	• ≤ 2 episodes between 2 feeds or in the previous 3 hours (if not fed)	• > 2 episodes between 2 feeds or in the previous 3 hours (if not fed)<br>• Bilious vomiting/hematemesis
Gastric residual volumes (GRVs)[a]	• GRV < 100% of previous feed (bilious or with hematic fragments)	• Hematic/fecaloidal GRV<br>• GRV ≥ 100% of previous feed
Alvus	• Mucous stools	• Hematic stools
Cardiorespiratory (CR) events	• ≥ 3 CR events[b]/h	• ≥ 1 extreme CR events[c]

0-1 minor criteria:	-	continue enteral feeding with increments as per protocol (max 30 mL/Kg/day)
2 minor criteria:	-	stop increasing feeds, re-assess prior to the next feed and evaluate GRV if not done before;
	-	if 2 minor criteria in at least 2 consecutive evaluations consider reducing volume of feed
1 major criterion or 3 minor criteria:	-	interrupt enteral feeding and re-assess prior to the next feed

a. The evaluation of gastric residual volumes is elective and according to the protocol of each research unit. Gastric residual volumes are considered pathological according to minor and major criteria.

b. CR events were defined as episodes of apnea lasting more than 20 seconds or over 5 seconds if followed by desaturation or bradycardia, episodes of desaturation with blood oxygen saturation below 80%, and episodes of bradycardia with heart rate below 80 beats per minute.

c. Extreme CR events were defined as CR events requiring resuscitation.

Table 2. Abdominal distension score

	Score
Abdomen is not distended	0
Abdomen is distended but not tense	1
Abdomen is distended and tense, responsive to gastric detension/rectal stimulation	2
Abdomen is distended and tense, not responsive to gastric detension/rectal stimulation	3

Data on respiratory support, nutrition, growth and overall clinical status will be collected from enrollment to discharge. According to an intention-to-treat model each patient will be monitored whatever the occurring clinical events, including the failure of the modality of respiratory support assigned at enrollment. Death or transfer to another hospital before reaching of full enteral feeding are the only reasons for a patient to drop the study.

All data to be collected will be obtained from the clinical records. Data will be recorded on a common database available on the ENTARES website and specifically designed for this study. Access to the database will be password protected and data will be entered by the local principal investigator. Participants will be identified by trial number only.

All data recorded throughout the study period are listed in Table 3.

Table 3. Data recorded during the study period.

Ventilation/respiration parameters [a]

- Respiratory support technique
- PEEP/CPAP (cmH_2O)
- PIP (cmH_2O)
- Flow (L/min)
- FiO_2 (%)
- Respiratory rate (acts/min)
- Transcutaneous O_2 blood saturation ($SatO_{2\ TC}$ %)
- Capillary/arterial blood gas test
- Episodes of apnea (> 20 s or > 5 s if followed by bradycardia/desaturation), bradycardia (HR ≤ 80 bpm) and desaturation ($SatO_{2\ TC}$ ≤ 80%)/day
- Silverman score

Feeding parameters [b]

- Parenteral nutrition intake (mL/Kg/day)
- Enteral nutrition intake (mL/Kg/day)
- Total caloric intake (Kcal/Kg/day)
- Type of milk: human milk or formula
- Modality of feeding (bolus, gavage, continuous feeding)
- Modality of fortification (if any: type of fortifier; standard, target or adjustable fortification)
- Enteral feeding interruptions (episodes/day)
- Not given feeds (episodes/day)
- Pathologic gastric residual volumes (episodes/day)
- Vomits and/or regurgitations (episodes/day)
- Abdominal distention (medium score/day)

Auxological parameters [c]

- Weight (g)
- Length (cm)
- Cranial circumference (cm)

Overall health status parameters[d]

- Patent ductus arteriosus
- Intraventricular hemorrhage
- Leukomalacia
- Retinopathy of prematurity
- Pneumothorax
- Blood transfusion
- NEC
- Intestinal perforation

[a.] Ventilation/respiration parameters will be recorded at enrollment, at achievement of half enteral feeding and full enteral feeding, at the beginning of oral feeding, at achievement of full oral feeding and at any change in respiratory assistance strategy. Apnea monitoring will extent until any respiratory support is needed (except for O2 supplementation per nasal cannula)

[b.] Feeding parameters will be recorded daily until full enteral feeding is achieved, at the beginning of oral feeding, at achievement of full oral feeding and at any change in respiratory assistance strategy.

c. Auxological parameters will be recorded at the time of enrollment, upon achieving half enteral feeding and full enteral feeding and at discharge.

d. Relevant clinical events/diagnosis will be recorded from enrollment until discharge

Outcomes

The primary outcome of the study is the time need to reach full enteral feeding (FEF), defined as an enteral intake of 150 mL/kg/die. Secondary outcomes are listed in Table 4.

Table 4. Secondary outcomes

– Time to reach half enteral feeding (HEF), defined as an enteral intake of 75 ml/kg/die (days)
– Interruptions of enteral feeding (episodes/day)
– Not given feeds (episodes/day)
– Pathologic gastric residual volumes (episodes/day)
– Vomits and/or regurgitations (episodes/day)
– Abdominal distention (mean score/day)
– Beginning of oral feeding (post-menstrual age)
– Time to reach full oral feeding (number of days)
– Post-menstrual age at full oral feeding (weeks)
– Weight growth (Δ z-score)
– Duration of the respiratory support assigned at randomization (days)
– Total duration of respiratory support need (days)
– Failure of the respiratory support assigned at randomization (yes/no)
– Length of hospital stay (days)
– Duration of central venous catheter (days)
– Clinical events and complications (NEC, bowel perforation, pneumothorax, BPD, PDA, ROP, IVH, PVL)
– Transfers to other hospitals or deaths before reaching full enteral feeding (number of patients)

HEF: half enteral feeding
NEC: necrotizing enterocolitis
BPD: bronchopulmonary dysplasia
PDA: patent ductus arteriosus
ROP: retinopathy of prematurity
IVH: intraventricular haemorrhage
PVL: periventricular leukomalacia

The design of the study is outlined in Figure 1.

Figure 1. Design of the study.

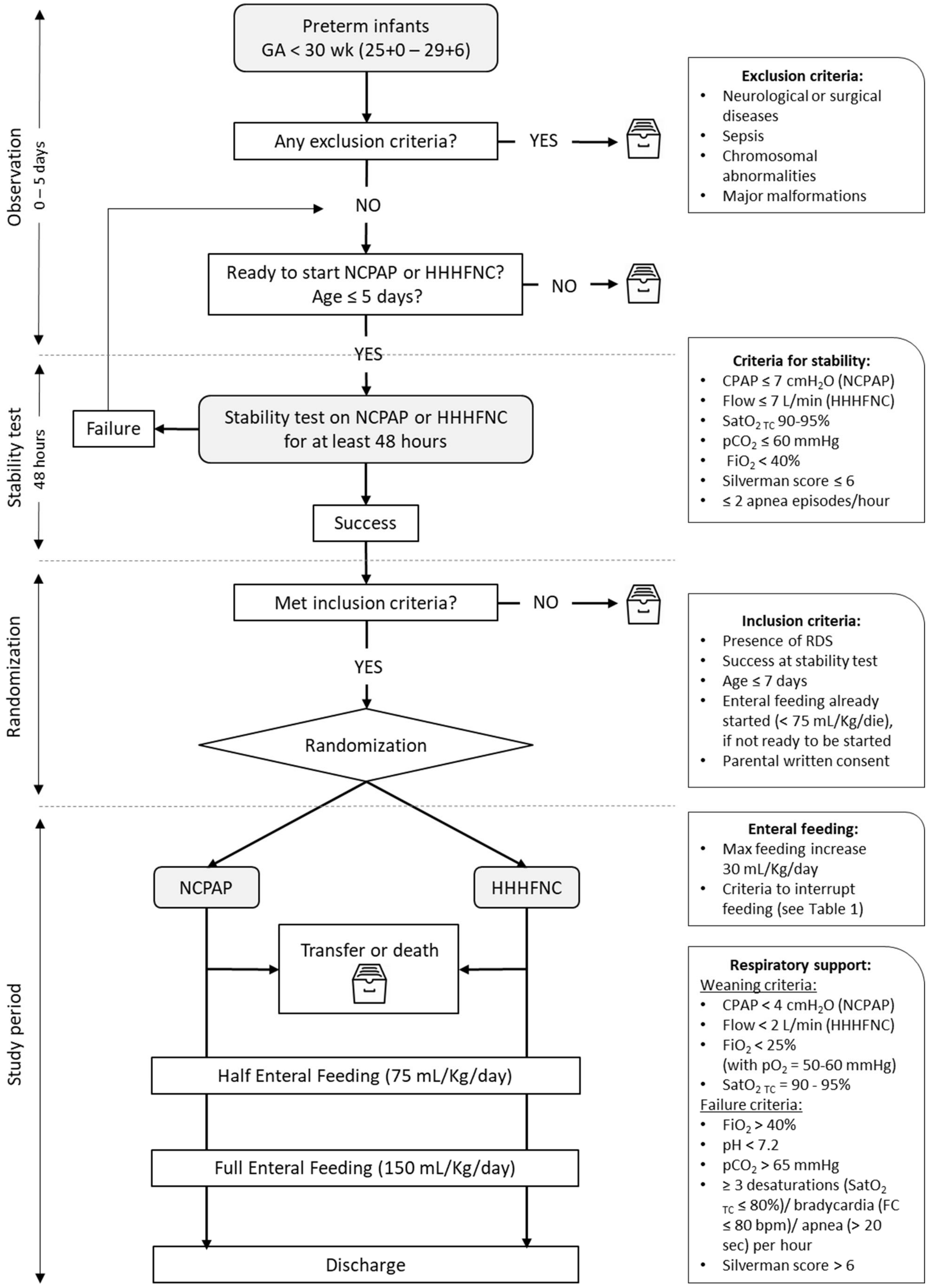

Statistical analysis plan

The Intention to Treat (ITT) population was defined as all the randomized infants, while the Per Protocol (PP) population was defined as all the infants who did not switch the arm assigned at randomization, not require invasive ventilation after randomization, and with data on full-enteral feeding (FEF); infants transferred or died before reaching FEF or with revoked consent will be excluded. The categorical variables will be summarised as frequencies (percent), while the continuous variables will be summarised as mean (Standard Deviation) or median [Inter Quartile Range] according to their distribution. SAS 9.4 software (SAS Institute, Cary, NC) will be used to process data and fit statistical models.

Primary outcome: time to FEF

The time to FEF will be computed as days from randomization to time to FEF (defined as an enteral intake of 150 mL/kg/die) achievement. The Kaplan Meier (KM) method will be performed to compare time to FEF between the two arms in the ITT population. Since the study was not blinded, casual switching of arms cannot be assumed. For this reason, the PP analysis will be performed using the Adjusted Kaplan Meier (AKM) method[9] with centre, intrauterine growth restriction (IUGR), and GA (weeks) as adjustment covariates. The difference between arms will be tested using the Log-Rank test. Supplemental analysis will be provided by GA classes (< 28 weeks, 28-29 weeks).

Secondary outcomes

Nutrition and comorbidity

The effect of respiratory support on nutritional outcomes and comorbidity will be estimated as risk of event comparing arms by Relative Risk (RR) with reference to NCPAP, using Poisson regression with robust error variance. The events considered for the analysis of nutritional outcomes are: ≥1 feeding interruption, severe abdominal distension (grade ≥2), ≥3 regurgitations or vomits in a day, ≥1 pathological gastric residual, and cardiorespiratory events.

NEC, pneumothorax (PNX), severe IVH[10], BPD, retinopathy of the prematurity (ROP), sepsis, and patent ductus arteriosus (PDA) requiring treatment will be considered in the analysis of comorbidity. In the PP population data will be adjusted by IUGR and GA. Centre will not be considered for adjustment as the events considered will be standardized by shared definitions or by the application of the study protocol in each participating NICUs.

Growth

The weight growth will be evaluated as the differences in grams from randomization to time to FEF per day: (weight at time to FEF-weight at randomization)/(weight at randomization*time to FEF).

The analysis was performed with a linear regression in which the weight growth are the dependent variable and the arm the independent variable. The analysis of PP population will be adjusted by IUGR, GA, centre, and age at age at randomization.

Respiratory

The time the assigned respiratory support is maintained, defined as time from randomization to the first change of respiratory support due to any cause, will be compared using a general linear model with log link and normal distribution. The estimates will be adjusted by centre and GA.

The frequencies of changed ventilator support and reason for the change will be evaluated by Fisher exact test, while the median fraction of inspired oxygen (FiO_2), blood oxygen saturation (SpO_2), and their ratio (SpO_2/FiO_2) will be compared by Kruskal-Wallis test.

Sample size

The sample size of 123 infants per arm was estimated to have 90% power to detect a 30% difference on the primary outcome (time to FEF, predicted to be 19.6 days from data from participating

neonatal intensive care units the year before the start of the study) using a two-sided Log-Rank test with a significance level of 0.05. Assuming 13% dropout, 282 eligible infants are expected to be enrolled.

QUALITY CONTROL AND QUALITY ASSURANCE PROCEDURES

Compliance to protocol:

Compliance will be defined as full adherence to protocol. Compliance with the protocol will be ensured by a number of procedures as described below.

ABBREVIATIONS

RDS: respiratory distress syndrome
BPD: bronchopulmonary dysplasia
NCPAP: nasal continuous positive air pressure
HHHFNC: heated humidified high-flow nasal cannula
NRS: non-invasive respiratory support
FI: feeding intolerance
NEC: necrotizing enterocolitis
NICU: neonatal intensive care unit
TC: transcutaneous
SAE: significant adverse event

REFERENCES

1. Silverman WA, Andersen DH. A controlled clinical trial of effects of water mist on obstructive respiratory signs, death rate and necropsy findings among premature infants. Pediatrics 1956;17(1):1–10.

2. Roehr CC, Yoder BA, Davis PG, Ives K. Evidence Support and Guidelines for Using Heated, Humidified, High-Flow Nasal Cannulae in Neonatology: Oxford Nasal High-Flow Therapy Meeting, 2015. Clin Perinatol 2016;43(4):693–705.

3. Yoder BA, Manley B, Collins C, et al. Consensus approach to nasal high-flow therapy in neonates. J Perinatol Off J Calif Perinat Assoc 2017;

4. Maas C, Franz AR, von Krogh S, Arand J, Poets CF. Growth and morbidity of extremely preterm infants after early full enteral nutrition. Arch Dis Child Fetal Neonatal Ed 2018;103(1):F79–81.

5. Martin RJ, Fanaroff AA, Walsh MC. Fanaroff and Martin's neonatal-perinatal medicine: diseases of the fetus and infant. 2015.

6. Lucchini R, Bizzarri B, Giampietro S, De Curtis M. Feeding intolerance in preterm infants. How to understand the warning signs. J Matern-Fetal Neonatal Med Off J Eur Assoc Perinat Med Fed Asia Ocean Perinat Soc Int Soc Perinat Obstet 2011;24 Suppl 1:72–4.

7. Li Y-F, Lin H-C, Torrazza RM, Parker L, Talaga E, Neu J. Gastric residual evaluation in preterm neonates: a useful monitoring technique or a hindrance? Pediatr Neonatol 2014;55(5):335–40.

8. Kaur A, Kler N, Saluja S, et al. Abdominal circumference or gastric residual volume as measure of feed intolerance in VLBW infants. J Pediatr Gastroenterol Nutr 2015;60(2):259–63.

9. Xie J, Liu C. Adjusted Kaplan-Meier estimator and log-rank test with inverse probability of treatment weighting for survival data. Stat Med 2005;24(20):3089–110.

10. Fanaroff AA, Stoll BJ, Wright LL, et al. Trends in neonatal morbidity and mortality for very low birthweight infants. Am J Obstet Gynecol 2007;196(2):147.e1-147.e8.

Summary of changes (protocol)

Background: unchanged
AIM: unchanged
METHODS:

- Study design and setting: unchanged
- Inclusion criteria: unchanged
- Exclusion criteria: unchanged
- Randomization: unchanged
- Monitoring and data collection
    - Initial setup: In the final version, the upper limit for HHHFNC was increased from 6 to 7 L/min.
    - Criteria to try weaning: unchanged
    - Failure criteria: unchanged
- Minimal standard criteria for enteral nutrition
    - maximum cut-off for feeding progression: unchanged
    - Criteria for the interruption of enteral feeding: Cardiorespiratory events was added in the final version (table 1)
    - Abdominal distension score: unchanged
- Data recorded during the study period
    - Primary outcome: unchanged
    - Secondary outcome: auxological parameters and the overall health status parameters were added in detail in the final version (table 3 and 4)
- Statistical analysis plan: in the final version the statistical analysis plan was explained in more detail and divided into sub-sections (primary outcome, Nutrition and comorbidity, Growth, Respiratory)
- Sample size: unchanged

Original statistical analysis plan

Statistical analysis and sample size Time to reach FEF, the primary outcome, will be analyzed by Kaplan and Meier survival analysis according to the intention-to-treat principle. The two arms will be compared with the log-rank Test.
Regarding secondary outcomes, the time to reach half enteral feeding and time to reach full oral feeding will be estimated by Kaplan and Meier analysis, the failure of the respiratory support assigned at randomization will be compared using Fischer's exact test, and the other secondary outcomes will be estimated using appropriate generalized linear models. This will be a single-blind trial where the blinded person will be the statistician. The sample size of 123 infants per arm was estimated to have 90% power to detect a 30% difference on the primary outcome (time to FEF, predicted to be 19.6 days from data from participating neonatal intensive care units the year before the start of the study) using a two-sided Log-Rank test with a significance level of 0.05. Assuming 13% dropout, 282 eligible infants are expected to be enrolled.

Final statistical analysis plan

The Intention to Treat (ITT) population was defined as all the randomized infants, while the Per Protocol (PP) population was defined as all the infants who did not switch the arm assigned at randomization, not require invasive ventilation after randomization, and with data on full-enteral feeding (FEF); infants transferred or died before reaching FEF or with revoked consent will be excluded. The categorical variables will be summarised as frequencies (percent), while the continuous variables will be summarised as mean (Standard Deviation) or median [Inter Quartile Range] according to their distribution. SAS 9.4 software (SAS Institute, Cary, NC) will be used to process data and fit statistical models.

Primary outcome: time to FEF

The time to FEF will be computed as days from randomization to time to FEF (defined as an enteral intake of 150 mL/kg/die) achievement. The Kaplan Meier (KM) method will be performed to compare time to FEF between the two arms in the ITT population. Since the study was not blinded, casual switching of arms cannot be assumed. For this reason, the PP analysis will be performed using the Adjusted Kaplan Meier (AKM) method[9] with centre, intrauterine growth restriction (IUGR), and GA (weeks) as adjustment covariates. The difference between arms will be tested using the Log-Rank test. Supplemental analysis will be provided by GA classes (< 28 weeks, 28-29 weeks).

Secondary outcomes

Nutrition and comorbidity

The effect of respiratory support on nutritional outcomes and comorbidity will be estimated as risk of event comparing arms by Relative Risk (RR) with reference to NCPAP, using Poisson regression with robust error variance. The events considered for the analysis of nutritional outcomes are: ≥1 feeding interruption, severe abdominal distension (grade ≥2), ≥3 regurgitations or vomits in a day, ≥1 pathological gastric residual, and cardiorespiratory events.

NEC, pneumothorax (PNX), severe IVH[10], BPD, retinopathy of the prematurity (ROP), sepsis, and patent ductus arteriosus (PDA) requiring treatment will be considered in the analysis of comorbidity. In the PP population data will be adjusted by IUGR and GA. Centre will not be considered for adjustment as the events considered will be standardized by shared definitions or by the application of the study protocol in each participating NICUs.

Growth

The weight growth will be evaluated as the differences in grams from randomization to time to FEF per day: (weight at time to FEF-weight at randomization)/(weight at randomization*time to FEF).

The analysis was performed with a linear regression in which the weight growth are the dependent variable and the arm the independent variable. The analysis of PP population will be adjusted by IUGR, GA, centre, and age at age at randomization.

Respiratory

The time the assigned respiratory support is maintained, defined as time from randomization to the first change of respiratory support due to any cause, will be compared using a general linear model with log link and normal distribution. The estimates will be adjusted by centre and GA.

The frequencies of changed ventilator support and reason for the change will be evaluated by Fisher exact test, while the median fraction of inspired oxygen (FiO_2), blood oxygen saturation (SpO_2), and their ratio (SpO_2/FiO_2) will be compared by Kruskal-Wallis test.

Sample size

The sample size of 123 infants per arm was estimated to have 90% power to detect a 30% difference on the primary outcome (time to FEF, predicted to be 19.6 days from data from participating neonatal intensive care units the year before the start of the study) using a two-sided Log-Rank test with a significance level of 0.05. Assuming 13% dropout, 282 eligible infants are expected to be enrolled.

Summary of changes (analysis plan)

- Statistical analysis plan: in the final version the statistical analysis plan was explained in more detail and divided into sub-sections (primary outcome, Nutrition and comorbidity, Growth, Respiratory)
- Sample size: unchanged
