# Supplementary material for: Effect of Nasal Continuous Positive Airway Pressure vs Heated Humidified High-Flow Nasal Cannula on Feeding Intolerance in Preterm Infants With Respiratory Distress Syndrome: The ENTARES Randomized Clinical Trial
Source: JAMA Netw Open. 2023 Jul 12;6(7):e2323052. doi: 10.1001/jamanetworkopen.2023.23052 (PMC10339152; doi:10.1001/jamanetworkopen.2023.23052)
Supplement: Supplement 2. — eTable 1. Criteria for the Interruption of Enteral Feeding eTable 2. Abdominal Distension Score eTable 3. Minimal Standard Criteria for Respiratory Support eTable 4. Secondary Outcomes Statistical Analysis eTable 5. Characteristics of Censored Infants eFigure 1. Infants Distribution by Centre and Arm eFigure 2. Infants Distribution by Centre and GA eFigure 3. Time to Reach Full Enteral Feeding (150 mL/kg/day) in NCPAP and HHHFNC Arms by GA [file jamanetwopen-e2323052-s002.pdf]

# Supplemental Online Content

Cresi F, Maggiora E, Lista G, et al; ENTARES Study Group. Effect of nasal continuous positive airway pressure vs heated humidified high-flow nasal cannula on feeding intolerance in preterm infants with respiratory distress syndrome: the ENTARES randomized clinical trial. *JAMA Netw Open*. 2023;6(7):e2323052. doi:10.1001/jamanetworkopen.2023.23052

**eTable 1.** Criteria for the Interruption of Enteral Feeding

**eTable 2.** Abdominal Distension Score

**eTable 3.** Minimal Standard Criteria for Respiratory Support

**eTable 4.** Secondary Outcomes Statistical Analysis

**eTable 5.** Characteristics of Censored Infants

**eFigure 1.** Infants Distribution by Centre and Arm

**eFigure 2.** Infants Distribution by Centre and GA

**eFigure 3.** Time to Reach Full Enteral Feeding (150 mL/kg/day) in NCPAP and HHHFNC Arms by GA

This supplemental material has been provided by the authors to give readers additional information about their work.

© 2023 Cresi F et al. *JAMA Network Open.*

Abbreviation list

BPD: bronchopulmonary dysplasia

CI: confidence interval

DOL: days of life

FI: feeding intolerance

GA: gestational age

GRV: gastric residual volumes

HHHFNC: heated humidified high-flow nasal cannula

IVH: intraventricular haemorrhage

KM: Kaplan Meier

NCPAP: nasal continuous positive air pressure

NEC: necrotizing enterocolitis

NICU: neonatal intensive care unit

NRS: non-invasive respiratory support

RCT: randomized controlled trial

RDS: respiratory distress syndrome

RR: relative risk

© 2023 Cresi F et al. *JAMA Network Open.*

**eTable 1**. Criteria for the interruption of enteral feeding.

| | Minor Criteria | Major Criteria |
|---|---|---|
| Physical examination | • Abdominal distension<br>• Visible bowel ansa<br>• Abdominal distension responsive to gastric detension/rectal stimulation | • Dyschromic abdominal wall<br>• Abdominal distension not responsive to gastric detension/rectal stimulation<br>• Painful abdomen |
| Regurgitations/vomits | • ≤ 2 episodes between 2 feeds or in the previous 3 hours (if not fed) | • > 2 episodes between 2 feeds or in the previous 3 hours (if not fed)<br>• Bilious vomiting/hematemesis |
| Gastric residual volumes (GRVs)[a] | • GRV < 100% of previous feed (bilious or with hematic fragments) | • Hematic/fecaloidal GRV<br>• GRV ≥ 100% of previous feed |
| Alvus | • Mucous stools | • Hematic stools |
| Cardiorespiratory (CR) events | • ≥ 3 CR events[b]/h | • ≥ 1 extreme CR events[c] |
| 0-1 minor criteria: | continue enteral feeding with increments as per protocol (max 30 mL/Kg/day) | |
| 2 minor criteria: | - stop increasing feeds, re-assess prior to the next feed and evaluate GRV if not done before;<br>- if 2 minor criteria in at least 2 consecutive evaluations consider reducing volume of feed | |
| 1 major criterion or 3 minor criteria: | - interrupt enteral feeding and re-assess prior to the next feed | |

a. The evaluation of gastric residual volumes is elective and according to the protocol of each research unit. Gastric residual volumes are considered pathological according to minor and major criteria.

b. CR events were defined as episodes of apnea lasting more than 20 seconds or over 5 seconds if followed by desaturation or bradycardia, episodes of desaturation with blood oxygen saturation below 80%, and episodes of bradycardia with heart rate below 80 beats per minute.

c. Extreme CR events were defined as CR events requiring resuscitation.

© 2023 Cresi F et al. *JAMA Network Open.*

**eTable 2**. Abdominal distension score

| | **Score** |
|---|---|
| Abdomen is not distended | 0 |
| Abdomen is distended but not tense | 1 |
| Abdomen is distended and tense, responsive to gastric detension/rectal stimulation | 2 |
| Abdomen is distended and tense, not responsive to gastric detension/rectal stimulation | 3 |

© 2023 Cresi F et al. *JAMA Network Open.*

**eTable 3**. Minimal standard criteria for respiratory support.

| | |
|---|---|
| ***Suggested initial setup*** | CPAP between 5 and 7 $cmH_2O$ if on NCPAP and flow between 4 and 7 L/min if on HHHFNC; |
| | $FiO_2$ is set as to reach $pO_2$ = 50 - 60 mmHg (capillary/arterial blood gas test) and $SatO_2$ TC = 90 - 95%. |
| ***Criteria to try weaning*** | CPAP < 4 $cmH_2O$ if on NCPAP and flow < 2 L/min if on HHHFNC |
| | FiO2 < 25% to maintain pO2 = 50 - 60 mmHg (capillary/arterial blood gas test) and $SatO_2$ TC = 90 - 95%. |
| ***Failure criteria*** | $FiO_2$ > 40% |
| | pH < 7.2 |
| | $pCO_2$ > 65 mmHg |
| | ≥ 3 episodes of desaturations ($SatO_2$ TC ≤ 80%) per hour |
| | ≥ 3 episodes of apnea (> 20 sec) and/or bradycardia (FC ≤ 80 bpm) per hour |
| | Silverman score > 6 |

Abbreviations: CPAP, continuous positive air pressure; NCPAP, nasal continuous positive air pressure; HHHFNC, heated humidified high flow nasal cannula; $FiO_2$, fraction of inspired oxygen; $pO_2$, partial oxygen pressure; $SatO_2$TC, transcutaneous oxygen; $pCO_2$, partial Carbon dioxide pressure.

© 2023 Cresi F et al. *JAMA Network Open.*

**eTable 4**. Secondary outcomes statistical analysis.

| | **Model** |
|---|---|
| **Nutritional outcomes** | |
| At least one episode of feeding interruption<br>At least one episode of feeding interruption > 1 day<br>At least one episode of pathological GRV<br>At least three episodes of vomit and/or regurgitation in a day<br>Abdominal distension score ≥2<br>At least one episode of cardiorespiratory events | Poisson regression with robust error variance (reference=NCPAP) |
| Time to full oral feeding (days from randomization) | general linear model with log-link |
| Daily enteral increment | linear regression |
| **Growth** | |
| $\frac{\text{weight at time to FEF(g)} - \text{weight at recruitment(g)}}{\text{weight at recruitment (g)} \times \text{time to FEF(days)}} \times 1000$ | linear regression |
| **Comorbidities (occurred after randomization)** | |
| Bronco pulmonary dysplasia (BPD)<br>Retinopathy of the prematurity (ROP)<br>Sepsis<br>Patent ductus arteriosus (PDA) which required treatment | Poisson regression with robust error variance (reference=NCPAP) |
| **Length Of Stay (LOS)** | |
| Time from randomization to discharge (days) | general linear model with log-link |
| **Respiratory outcomes** | |
| $SatO_2/FiO_2$ ratio | Kruskal-Wallis test |
| Frequencies of arm changes | Fisher exact test |
| Time the respiratory support assigned at randomization was maintained | general linear model with log-link, adjusted by GA, centre |

© 2023 Cresi F et al. *JAMA Network Open.*

**eTable 5.** Characteristics of censored infants.

| | **NCPAP N=9 N (%)** | **HHHFNC N=10 N (%)** |
|---|---|---|
| Reason for censoring | | |
| *discharged with an enteral intake < 150 mL/kg/day* | 4 (44.4) | 5 (50.0) |
| *transferred* | 1 (11.1) | - |
| *dead* | 2 (22.2) | 2 (20.0) |
| *consent withdrawn* | - | 2 (20.0) |
| *others* | 2 (22.2) | 1 (10.0) |
| Respiratory supports at censoring | | |
| *more invasive support* | 1 (11.1) | 3 (30.0) |
| *no respiratory support* | 5 (55.6) | 4 (40.0) |
| *NCPAP* | 3 (33.3) | - |
| *HHHFNC* | - | 3 (30.0) |
| Comorbidity between randomization and censoring | | |
| *BPD* | 1 (11.1) | 1 (10.0) |
| *NEC* | 1 (11.1) | 1 (10.0) |
| *ROP* | - | 2 (20.0) |
| *Sepsi* | 4 (44.4) | 5 (50.0) |
| *PTX* | - | - |
| *IVH* | - | - |
| *PDA* | 3 (33.3) | 2 (20.0) |
| GA, median (IQR) | 27 (26-29) | 27 (26-29) |
| Time to censoring, median (IQR), days | 25 (11-58) | 32 (6-62) |
| Time at the first change of respiratory support, median (IQR), days | 4 (2-6) | 10.5 (2.5-14.5) |
| Enteral intake at censoring, median (IQR), mL/Kg/day | 32 (24-70) | 126 (30-150) |

Abbreviations: NCPAP, nasal continuous positive air pressure; HHHFNC heated humidified high flow nasal cannula; BPD, bronchopulmonary dysplasia; NEC, necrotizing enterocolitis; ROP, retinopathy of the prematurity; PTX, pneumothorax; IVH, intraventricular haemorrhage; PDA, patent ductus arteriosus; GA, gestational age.

© 2023 Cresi F et al. *JAMA Network Open.*

**eFigure 1.** Infants distribution by centre and arm.

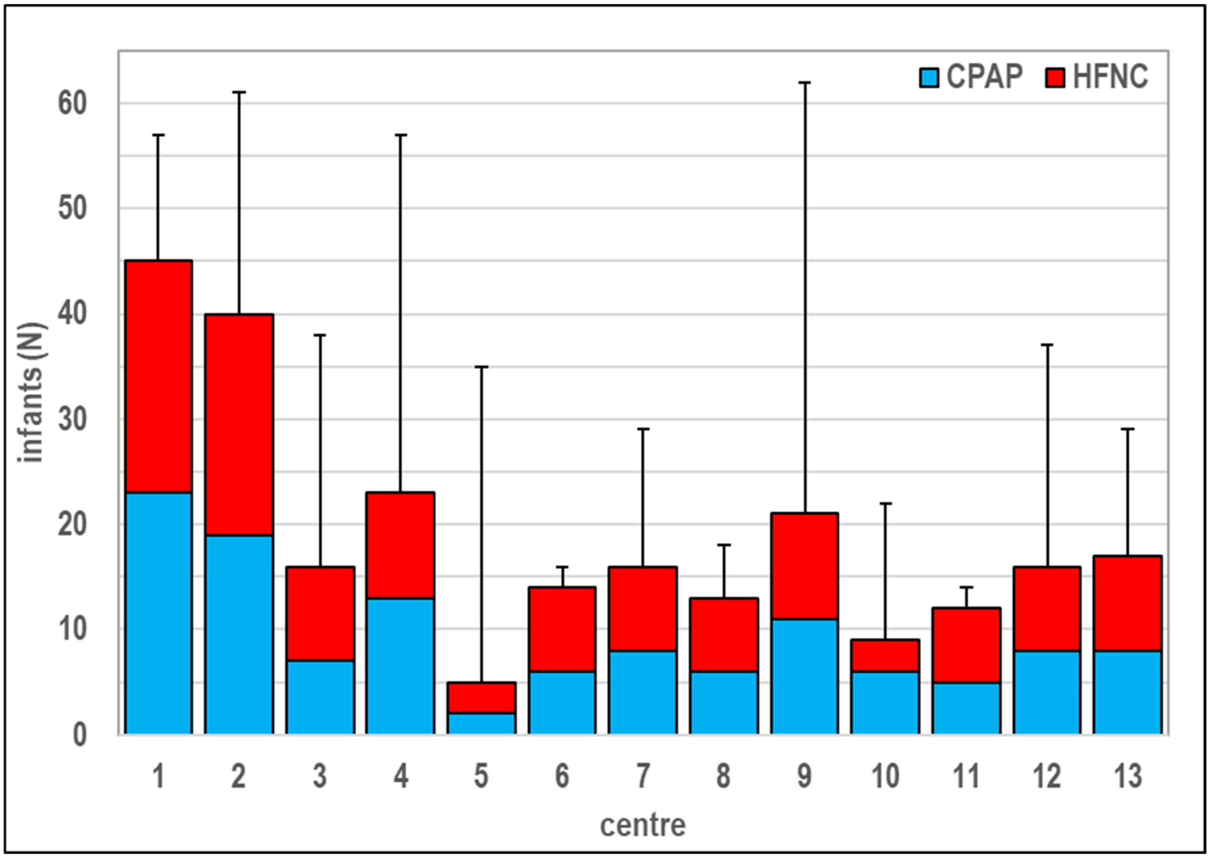


Abbreviations: NCPAP, nasal continuous positive air pressure; HHHFNC, heated humidified high flow nasal cannula.

Distribution of infants by centre and arm. The vertical bar represents the number of eligible infants.

© 2023 Cresi F et al. *JAMA Network Open.*

**eFigure 2.** Infant distribution by centre and GA.

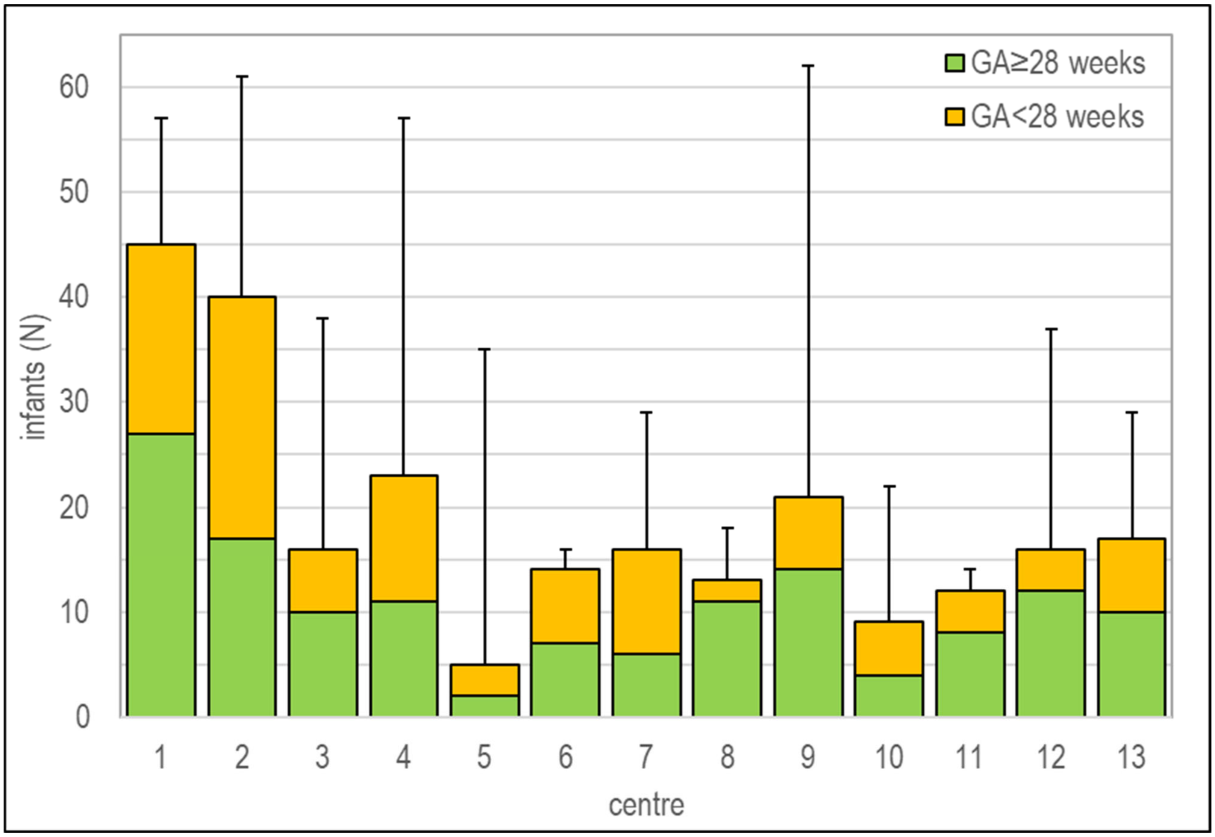


Abbreviations: GA, gestational age; NCPAP, nasal continuous positive air pressure; HHHFNC, heated humidified high flow nasal cannula.

Distribution of infants by centre and GA. The vertical bar represents the number of eligible infants.

© 2023 Cresi F et al. *JAMA Network Open.*

**eFigure 3.** Time to reach full enteral feeding (150 mL/kg/day) in NCPAP and HHHFNC arms by GA.

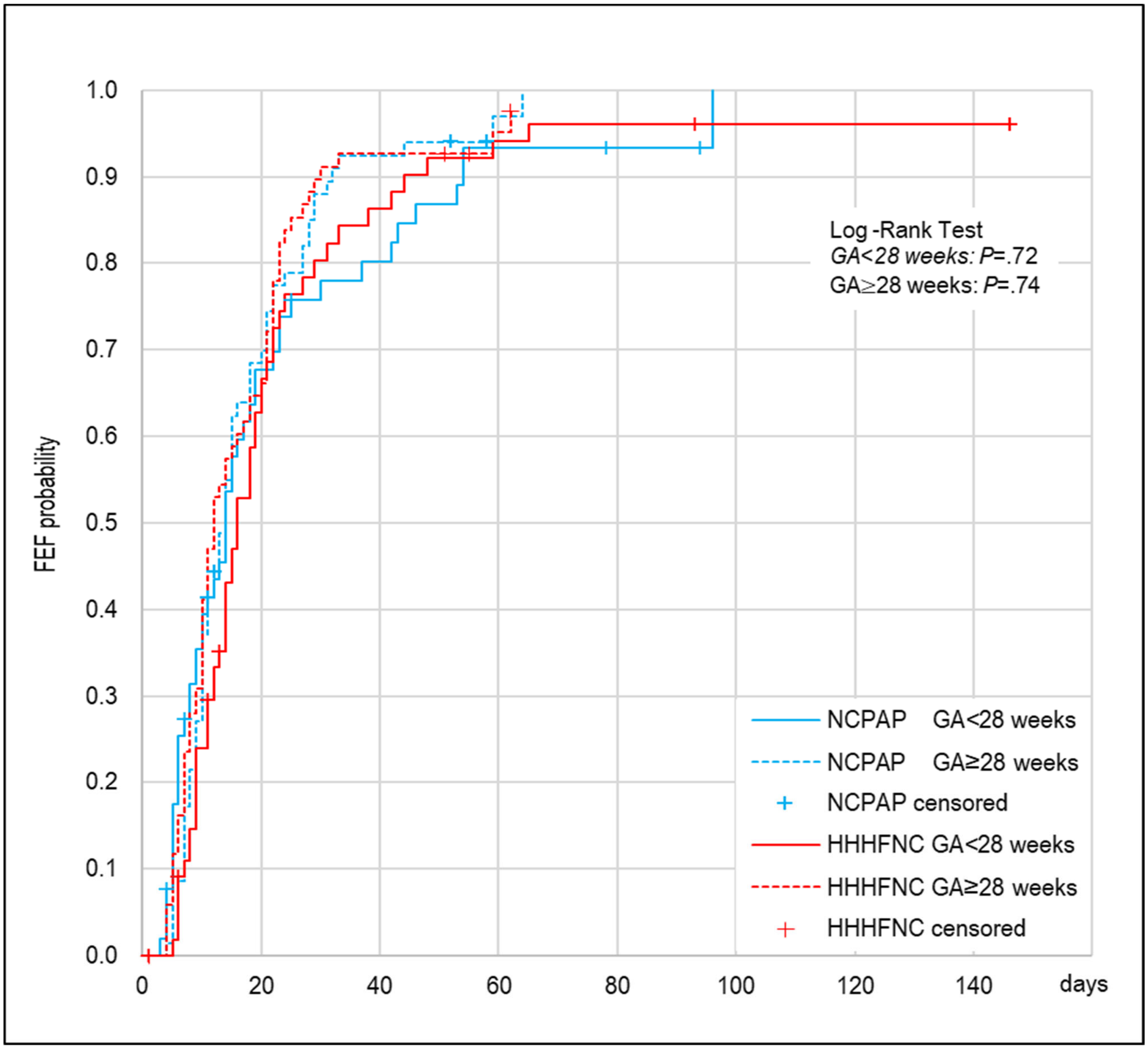


Abbreviations: FEF, full enteral feeding; NCPAP, nasal continuous positive air pressure; HHHFNC, heated humidified high flow nasal cannula; GA gestational age.

Kaplan Meier FEF probability estimates for NCPAP (blue) and HHHFNC (red) arm by class of GA (<28 weeks continuous lines, ≥28 weeks dotted lines). Symbol “+” indicates a censored observation.

© 2023 Cresi F et al. *JAMA Network Open.*
