# Supplementary material for: Effect of Nasal Continuous Positive Airway Pressure vs Heated Humidified High-Flow Nasal Cannula on Feeding Intolerance in Preterm Infants With Respiratory Distress Syndrome: The ENTARES Randomized Clinical Trial
Source: JAMA Netw Open. 2023 Jul 12;6(7):e2323052. doi: 10.1001/jamanetworkopen.2023.23052 (PMC10339152; doi:10.1001/jamanetworkopen.2023.23052)
Supplement: Supplement 3. — Nonauthor Collaborators [file jamanetwopen-e2323052-s003.pdf]

Supplemental Online Content: Nonauthor Collaborators
*First name, last name, and suffix (if applicable) are required and will appear in PubMed.

*Group Name(s): ENTARES Study Group							
***First Name and Middle Initial(s)**	***Last Name**	***Suffix (eg, Jr, III)**	Academic Degrees	Institution	Location (city, state/province, country)	Role or Contribution, eg, chair, principal investigator	Group (if more than 1 Group listed in the byline) and/or Subgroup (eg, Steering Committee)
Michela	Fiora		MD	Neonatology and Neonatal Intensive Care Unit, University of Turin	Torino, Italy	Clinical Investigator	
Sara	Colombo		MD	Neonatology and Neonatal Intensive Care Unit, University of Turin	Torino, Italy	Clinical Investigator	
Chiara	Peila		MD, PhD	Neonatology and Neonatal Intensive Care Unit, University of Turin	Torino, Italy	Clinical Investigator	
Mattia	Luciano		MSc	Neonatology and Neonatal Intensive Care Unit, University of Turin	Torino, Italy	Research Nurse Coordinator	
Fabio	Meneghin		MD	Neonatal Pathology and Neonatal Intensive Care Unit, Vittore-Buzzi Children Hospital	Milano, Italy	Principal Investigator	
Sara	Gatto		MD	Neonatal Pathology and Neonatal Intensive Care Unit, Vittore-Buzzi Children Hospital	Milano, Italy	Clinical Investigator	
Enrica	Lupo		MD	Neonatal Pathology and Neonatal Intensive Care Unit, Vittore-Buzzi Children Hospital	Milano, Italy	Clinical Investigator	
Serena	Elia		MD	Division of Neonatology, Careggi University Hospital of Florence	Firenze, Italy	Principal Investigator	
Martina	Ciarcià		MD	Division of Neonatology, Careggi University Hospital of Florence	Firenze, Italy	Clinical Investigator	
Fabio	Mosca		Professor	Fondazione IRCCS Ca' Granda Ospedale Maggiore Policlinico - Department of Clinical Sciences and Community Health, University of Milan	Milano, Italy	Chair	

Supplemental Online Content: Nonauthor Collaborators
*First name, last name, and suffix (if applicable) are required and will appear in PubMed.

*First Name and Middle Initial(s)	*Last Name	*Suffix (eg, Jr, III)	Academic Degrees	Institution	Location (city, state/province, country)	Role or Contribution, eg, chair, principal investigator	Group (if more than 1 Group listed in the byline) and/or Subgroup (eg, Steering Committee)
Anna	Orsi		MD	Fondazione IRCCS Ca' Granda Ospedale Maggiore Policlinico - Department of Clinical Sciences and Community Health, University of Milan	Milano, Italy	Principal Investigator	
Domenica	Mercadante		MD	Fondazione IRCCS Ca' Granda Ospedale Maggiore Policlinico - Department of Clinical Sciences and Community Health, University of Milan	Milano, Italy	Clinical Investigator	
Matilde	Amatruda		MD	Fondazione IRCCS Ca' Granda Ospedale Maggiore Policlinico - Department of Clinical Sciences and Community Health, University of Milan	Milano, Italy	Clinical Investigator	
Barbara	Tomasini		MD	U.O.C Terapia Intensiva Neonatale e Patologia Neonatale - Azienda Ospedaliera Universitaria Senese	Siena, Italy	Chair	
Sara	Cecchi		MD	U.O.C Terapia Intensiva Neonatale e Patologia Neonatale - Azienda Ospedaliera Universitaria Senese	Siena, Italy	Principal Investigator	
Pamela	Liuzzo Lasagna		MD	U.O.C Terapia Intensiva Neonatale e Patologia Neonatale - Azienda Ospedaliera Universitaria Senese	Siena, Italy	Clinical Investigator	
Stefano	Martinelli		MD	ASST Grande Ospedale Metropolitano Niguarda	Milano, Italy	Chair	
Laura	Ilardi		MD	ASST Grande Ospedale Metropolitano Niguarda	Milano, Italy	Principal Investigator	
Alice	Proto		MD	ASST Grande Ospedale Metropolitano Niguarda	Milano, Italy	Clinical Investigator	
Giovanni	Vento		Professor	Fondazione Policlinico Universitario A. Gemelli IRCCS, Dipartimento per le Scienze Della Salute Della Donna, del Bambino e di Sanità Pubblica	Roma, Italy	Chair	

Supplemental Online Content: Nonauthor Collaborators
*First name, last name, and suffix (if applicable) are required and will appear in PubMed.

***First Name and Middle Initial(s)**	***Last Name**	***Suffix (eg, Jr, III)**	Academic Degrees	Institution	Location (city, state/province, country)	Role or Contribution, eg, chair, principal investigator	Group (if more than 1 Group listed in the byline) and/or Subgroup (eg, Steering Committee)
Simonetta	Costa		MD	Fondazione Policlinico Universitario A. Gemelli IRCCS, Dipartimento per le Scienze Della Salute Della Donna, del Bambino e di Sanità Pubblica	Roma, Italy	Principal Investigator	
Francesca P	Fusco		MD	Fondazione Policlinico Universitario A. Gemelli IRCCS, Dipartimento per le Scienze Della Salute Della Donna, del Bambino e di Sanità Pubblica	Roma, Italy	Clinical Investigator	
Daniele	Farina		MD	Neonatology and Neonatal Intensive Care Unit, S. Anna Hospital – Città della Salute e della Scienza di Torino	Torino, Italy	Chair	
Maria F	Campagnoli		MD	Neonatology and Neonatal Intensive Care Unit, S. Anna Hospital – Città della Salute e della Scienza di Torino	Torino, Italy	Principal Investigator	
Tatiana	Boetti		MD	Neonatology and Neonatal Intensive Care Unit, S. Anna Hospital – Città della Salute e della Scienza di Torino	Torino, Italy	Clinical Investigator	
Elena	Boano		BSc	Neonatology and Neonatal Intensive Care Unit, S. Anna Hospital – Città della Salute e della Scienza di Torino	Torino, Italy	Research Nurse	
Andrea	Sannia		MD	Neonatal Intensive Care Unit, S. Croce e Carle Hospital	Cuneo, Italy	Chair	
Cinzia	De Maio		MD	Neonatal Intensive Care Unit, S. Croce e Carle Hospital	Cuneo, Italy	Principal Investigator	
Eleonora	Murizasco		MD	Neonatal Intensive Care Unit, S. Croce e Carle Hospital	Cuneo, Italy	Clinical Investigator	
Francesco	Raimondi		Professor	Neonatologia, Dipartimento Scienze Mediche Traslazionali. Università Federico II	Napoli, Italy	Chair	

Supplemental Online Content: Nonauthor Collaborators
*First name, last name, and suffix (if applicable) are required and will appear in PubMed.

***First Name and Middle Initial(s)**	***Last Name**	***Suffix (eg, Jr, III)**	Academic Degrees	Institution	Location (city, state/province, country)	Role or Contribution, eg, chair, principal investigator	Group (if more than 1 Group listed in the byline) and/or Subgroup (eg, Steering Committee)
Letizia	Capasso		MD	Neonatologia, Dipartimento Scienze Mediche Traslazionali. Università Federico II	Napoli, Italy	Principal Investigator	
Serena	Salomè		MD	Neonatologia, Dipartimento Scienze Mediche Traslazionali. Università Federico II	Napoli, Italy	Clinical Investigator	
Massimo	Agosti		Professor	Neonatologia, Terapia Intensiva Neonatale, Azienda Ospedaliero-Universitaria Asst-Settelaghi, Università degli Studi dell’Insubria	Varese, Italy	Chair	
Laura	Morlacchi		MD	Neonatologia, Terapia Intensiva Neonatale, Azienda Ospedaliero-Universitaria Asst-Settelaghi, Università degli Studi dell’Insubria	Varese, Italy	Principal Investigator	
Simona	Perniciaro		MD	Neonatologia, Terapia Intensiva Neonatale, Azienda Ospedaliero-Universitaria Asst-Settelaghi, Università degli Studi dell’Insubria	Varese, Italy	Clinical Investigator	
Nicola	Laforgia		Professor	Department of Interdisciplinary Medicine - Section of Neonatology and Neonatal Intensive Care Unit, "Aldo Moro" University of Bari	Bari, Italy	Chair	
Mariella	Baldassarre		MD	Department of Interdisciplinary Medicine - Section of Neonatology and Neonatal Intensive Care Unit, "Aldo Moro" University of Bari	Bari, Italy	Principal Investigator	
Manuela	Capozza		MD	Department of Interdisciplinary Medicine - Section of Neonatology and Neonatal Intensive Care Unit, "Aldo Moro" University of Bari	Bari, Italy	Clinical Investigator	
Mauro	Stronati		Professor	Neonatal Unit and Neonatal Intensive Care Unit, Fondazione IRCCS Policlinico San Matteo	Pavia, Italy	Chair	
Elisa	Civardi		MD	Neonatal Unit and Neonatal Intensive Care Unit, Fondazione IRCCS Policlinico San Matteo	Pavia, Italy	Principal Investigator	

Supplemental Online Content: Nonauthor Collaborators

*First name, last name, and suffix (if applicable) are required and will appear in PubMed.

***First Name and Middle Initial(s)**	***Last Name**	***Suffix (eg, Jr, III)**	Academic Degrees	Institution	Location (city, state/province, country)	Role or Contribution, eg, chair, principal investigator	Group (if more than 1 Group listed in the byline) and/or Subgroup (eg, Steering Committee)
Francesca	Garofoli		MD	Neonatal Unit and Neonatal Intensive Care Unit, Fondazione IRCCS Policlinico San Matteo	Pavia, Italy	Clinical Investigator	
Luca	Maggio		MD	Neonatology and Neonatal Intensive Care Unit, AO San Camillo Forlanini	Roma, Italy	Advisor	
Luigi	Corvaglia		Professor	Neonatal Intensive Care Unit, IRCCS Azienda Ospedaliero-Universitaria di Bologna; Department of Medical and Surgery Sciences, University of Bologna	Bologna, Italy	Advisor	
