# Supplementary material for: Effect of Nasal Continuous Positive Airway Pressure vs Heated Humidified High-Flow Nasal Cannula on Feeding Intolerance in Preterm Infants With Respiratory Distress Syndrome: The ENTARES Randomized Clinical Trial
Source: JAMA Netw Open. 2023 Jul 12;6(7):e2323052. doi: 10.1001/jamanetworkopen.2023.23052 (PMC10339152; doi:10.1001/jamanetworkopen.2023.23052)
Supplement: Supplement 4. — Data Sharing Statement [file jamanetwopen-e2323052-s004.pdf]

Data Sharing Statement

Cresi. Effect of Nasal Continuous Positive Airway Pressure vs Heated Humidified High-Flow Nasal Cannula on Feeding Intolerance in Preterm Infants With Respiratory Distress Syndrome. *JAMA Netw Open*. Published July 12, 2023. doi:10.1001/jamanetworkopen.2023.23052

Data
Data available: Yes
Data types: Deidentified participant data
How to access data: francesco.cresi@unito.it
When available: With publication

Supporting Documents
Document types: None

Additional Information
Who can access the data: researchers whose proposed use of the data has been approved
Types of analyses: future ancillary studies and meta-analyses approved by the authors (ENTARES study group)
Mechanisms of data availability: with investigator support
